# Implementation bottlenecks of near point of care HIV viral load monitoring for children and young people in Tanzania: A Qualitative Study

**DOI:** 10.1371/journal.pone.0351304

**Published:** 2026-06-12

**Authors:** Perry Msoka, Alan Mtenga, Rehema Maro, Rhoda Akello, Iraseni Ufoo Swai, Blandina T. Mmbaga, Ria Reis, Marion Sumari-de Boer

**Affiliations:** 1 Kilimanjaro Clinical Research Institute, Moshi, Tanzania; 2 Amsterdam Institute for Social Science Research, Amsterdam, the Netherlands; 3 Amsterdam Institute for Global Health and Development, Amsterdam, the Netherlands; 4 National Institute for Medical Research, Muhimbili, Dar es Salaam, Tanzania; 5 Amsterdam UMC, Location AMC, Amsterdam, the Netherlands; 6 School of Medicine, KCMC University, Moshi, Tanzania; 7 Kilimanjaro Christian Medical Centre, Moshi, Tanzania; 8 The Children’s Institute, University of Cape Town, Cape Town, South Africa; 9 Department of Epidemiology & Biostatistics, KCMC University, Moshi, Tanzania; University of Zimbabwe Faculty of Medicine: University of Zimbabwe College of Health Sciences, ZIMBABWE

## Abstract

**Background:**

Near point-of-care (n)POC Human Immunodeficiency Virus (HIV) viral load (VL) monitoring, consisting of VL testing in laboratories close to HIV treatment facilities, improves turnaround time from sampling to result. Other benefits of using nPOC monitoring are reduced laboratory workload, and limited loss of results, all leading to improved clinic retention and treatment adherence. However, the specific implementation bottlenecks are still unclear. This study aims to investigate the bottlenecks in the implementation of nPOC HIV VL monitoring among children and young people (ages 0–24 years) living with HIV, as experienced by healthcare workers (HCWs) in Tanzania.

**Methods:**

We conducted observations in clinics and in-depth interviews with HCWs from January 2023 to January 2024 at Tanzanian intervention sites within the East Africa Point-of-Care Viral Load Monitoring (EAPOC-VL) study. The EAPOC-VL study was conducted in four countries in East Africa (Tanzania, Kenya, Rwanda, Uganda). It was a cluster-randomised controlled trial, in which participants were tested at three time points (months 0, 6, and 12). We purposively selected 25 HCWs involved in implementing nPOC at the intervention sites for in-depth interviews. We interviewed HCWs at baseline (month 0) and after the initiation of nPOC HIV VL monitoring (months 1 and 6). We conducted deductive thematic framework analysis using the Measurement Instrument for Determinants of Innovations (MIDI), to which an inductive approach was added to identify facilitators and barriers across intervention, provider, organisational, social and political contexts. We used NVivo 12 to organise the data.

**Results:**

A total of 75 interviews were conducted among 43 HCWs across 3 time points: 33 at baseline (T0), 25 at month 1 (T1; 19 participants from T0 and 6 new participants) and 17 at month 6 follow up (T2;6 participants from T1, 7 returning participants from T0 and 4 new participants). Observations and interviews showed that nPOC HIV VL testing improved result turnaround time and enabled same-day counselling, which motivated both HCWs and clients. This data showed that knowledge, confidence, and adherence to procedures after training. Near POC, HIV VL was supported by compatibility with existing practices, strong teamwork, and management commitment. However, challenges included clients waiting at the clinic for over two hours to receive their results, the scarcity of resources, such as rooms and electricity, and staff shortages. Finally, delays were observed when samples had to be transported to nearby laboratories.

**Conclusion:**

Near POC HIV VL monitoring shortens turnaround times and enables immediate counselling. To maximise these benefits, there is a need to prioritise investment in staff training, infrastructure, improving sample handling/turnaround time and guideline alignment. Developing these areas will enhance service delivery and allow for improved outcomes among children and young people living with HIV.

## Background

The joint United Nations Programme on HIV and AIDS (UNAIDS) reported that in 2023, 39.9 million people worldwide had HIV, with low and middle-income countries like Tanzania bearing most of the burden [1]. The Tanzanian HIV Impact Survey(2023) estimated an HIV incidence of 0.18% among adults aged 15 and above, equivalent to about 60,000 new infections yearly [2]. In addition, the Tanzania Prevention of Mother-to- Child Transmission report showed that 3,800 children aged 0–14 years died with HIV in 2023 [3].

UNAIDS’ goal is to end the HIV/AIDS pandemic by 2030 through the 95-95-95 targets, ensuring that 95% of all people living with HIV (PLHIV) are aware of their status, 95% of those diagnosed receive sustained antiretroviral treatment (ART), and 95% of those on ART achieve viral suppression [4]. While Tanzania has made progress in testing and treatment coverage, achieving viral suppression (the third 95) remains a challenge, particularly among children and young people(aged 0–24 years), who face adherence barriers linked to stigma, psychosocial factors and dependence on caregivers [5–7].

The World Health Organisation (WHO) 2021 guideline categorises HIV viral load (VL) into three groups: virological failure (>1000 copies/mL), low-level viremia (>50 and ≤ 1000 copies/mL), and ≤50 copies/mL (undetectable; target not detected) [8]. Many national programs use > 1000 copies/ML to define virologic failure for programmatic reporting, while clinicians often consider VL < 50 copies/ML as undetectable in clinical practice. To monitor these levels, WHO recommends viral load (VL) testing at six months after ART initiation, again at 12 months, and annually thereafter [9]. However, the existing standard approach of centralised VL testing often faces delays due to transport, sample errors, and the misplacement of results, limiting timely care [10]. To overcome the delay challenges, point-of-care (POC) and near point-of-care(nPOC) HIV VL testing have been introduced. POC HIV VL monitoring entails testing at the point where healthcare is provided, i.e., within the clinic itself, using cartridge-based machines performed by non-laboratory skilled staff, with results available within approximately 90 minutes [9]. Near POC HIV VL monitoring, by contrast, entails testing in a laboratory near but not within the treatment facilities, requiring sample transport but still providing results faster than centralised laboratory testing [11]. Despite these advantages, implementation challenges persist. Studies from Uganda and Kenya highlighted barriers such as inadequate training, staff shortages, increased workload, power interruptions and equipment reliability issues [12–14].

Since nPOC HIV VL is a new recommended way of delivering fast and efficient VL testing, there is limited evidence or monitoring in Tanzania regarding the specific implementation bottlenecks it entails. The present study was nested within the East Africa Point-of-Care (EAPOC-VL) study, a cluster-randomised controlled trial and implementation study conducted in four East African countries: Tanzania, Kenya, Rwanda, and Uganda. In the main study, clusters, each consisting of one or more HIV treatment centres, were randomised to either the intervention arm, that was using nPOC HIV VL monitoring, or to the control arm, that was using conventional centralised VL testing. The study evaluated the effect of nPOC HIV VL monitoring compared to standard centralized monitoring on viral load suppression by taking blood samples at months 6 and 12 of follow-up. We conducted observations in the clinic and in-depth interviews with HCWs at intervention sites of the East Africa Point of Care (EAPOC-VL) study. The substudy focused on exploring the experience of HCWs regarding the implementation bottlenecks of nPOC HIV VL monitoring among children and young people with HIV in Tanzania. The objectives were (1) to investigate factors that contribute to good implementation of nPOC HIV VL monitoring and (2) to evaluate challenges faced by HCWs in implementing nPOC HIV VL monitoring.

## Methodology

### Study design and population

We conducted an exploratory qualitative study nested within the EAPOC VL trial (ClinicalTrials.gov identifier: NCT05048472; https://clinicaltrials.gov/study/NCT05048472) from January 2023 to January 2024. The study employed a combined longitudinal and cross-sectional design, which allowed follow-up of participants over time while also recruiting new participants at later time points.

Participants in the study included laboratory technicians, nurses, doctors, clinical officers, data clerks, pharmacists, peer educators, and counsellors. Recruitment of participants began on 11 February 2023 and ended on 28 January 2024. Data were collected through semi-structured, in-depth interviews with HCWs who had direct contact with clients in care and treatment centres (CTCs), as well as semi-structured observations.

### Study area and setting

We conducted this study in the Kilimanjaro region in the north of Tanzania and the Dar es Salaam region on the east coast of Tanzania. The study was conducted at five CTCs across the country, including a Referral Hospital (Mawenzi in Kilimanjaro), two district hospitals (Sinza in Dar es Salaam and Hai in Kilimanjaro), and two health centres (Pasua and Majengo, both in Kilimanjaro). Two sites (Sinza and Hai) performed nPOC HIV VL tests at on-site hospital laboratories with no transport required, while three sites (Mawenzi, Pasua, and Majengo) transported samples by boda-boda (motorcycle courier) to the off-site Kilimanjaro Clinical Research Laboratory for testing. All testing was conducted using GeneXpert machines, which can process up to four samples at a time and provide results in approximately 90 minutes. All interviews were conducted in private rooms within the clinics to ensure confidentiality and no third parties were present during interviews.

### Sampling of participants

For the interviews, the HCWs were selected purposively from those involved in implementing nPOC HIV VL monitoring at the intervention sites; that is, HCWs (1) attending PLWHIV at CTCs in one of the five selected health facilities, (2) who were involved in VL monitoring, (3) who were part of the EAPOC-VL study, (4) who were providing healthcare treatment and advice based on formal training and experience, such as doctors, nurses, and laboratory technicians and (5) who were not formally trained in providing healthcare, like data managers, peer educators and counsellors. We aimed for maximum variation, including a broad range of the professional cadre involved in implementing nPOC HIV VL monitoring. The chief physician of the selected intervention health facilities assisted in identifying locally practising HCWs who were currently or were previously involved in HIV client diagnostics and management and staff involved in the EAPOC-VL study. We initially planned to conduct an average of 25 interviews (five per site) across T0, T1, and T2, for a total of 75 interviews, based on the saturation principle [15,16]. All HCWs invited agreed to participate and no participant withdrew from the study. However, not all HCWs enrolled at baseline were available for follow-up interviews at T1 and T2 due to annual leave, staff transfers, and changing clinical responsibilities. To ensure sufficient data, new participants were recruited consistently with the study’s combined longitudinal and cross-sectional design.

### Theoretical framework

To investigate implementation bottlenecks, we focused on the feasibility of nPOC HIV VL monitoring, and we used the MIDI theoretical framework [17]. The MIDI framework is designed to help implementation researchers improve their understanding of the critical determinants that may affect the implementation of an intervention in order to better target the implementation strategy. The framework consists of 29 subdomain constructs divided into four groups of determinants (domains) associated with (1) the intervention, (2) the provider, (3) the implementing organisation, and (4) the social, political, and cultural context. The operationalisation of each domain and its subdomains is presented in Table 1.

**Table 1:** Items and constructs of the adapted MIDI for nPOC HIV VL monitoring.

Domain	Subdomain	Item/Construct	Operationalisation
The Intervention	Procedural Clarity	1	The extent to which nPOC HIV VL monitoring is described in clear steps/procedures
	Correctness	2	The degree to which nPOC HIV VL monitoring is based on factual, correct knowledge
	Completeness	3	The degree to which the activities described in nPOC HIV VL monitoring are carried out completely
	Complexity	4	The degree to which nPOC HIV VL monitoring is complex
	Compatibility	5	The degree to which nPOC HIV VL monitoring is compatible with the values and working methods in place
	Observability	6	The visibility of the outcomes for HCWs; for example, whether the outcomes of the laboratory tests are clear to HCWs
	Relevance for the client	7	The degree to which the HCWs believe nPOC HIV VL monitoring is relevant for his/her client
The Provider	Personal benefits	8a	The degree to which using nPOC HIV VL monitoring has advantages for the HCWs themselves
	Personal drawback	8b	The degree to which using nPOC HIV VL monitoring has disadvantages for the HCWs themselves
	Outcome expectations	9	The perceived probability and importance of achieving the HCWs’ objectives as intended by nPOC HIV VL monitoring
	Professional obligation	10	The degree to which nPOC HIV VL monitoring fits with the tasks for which HCWs feel responsible when doing their work
	Client satisfaction	11	The degree to which the HCWs expect clients to be satisfied with nPOC HIV VL monitoring
	Client cooperation	12	The degree to which HCWs expect clients to cooperate with nPOC HIV VL monitoring
	Social support	13	The support experienced or expected by the user from important social referents relating to the use of nPOC HIV VL monitoring
	Descriptive norm	14	Colleagues’ observed behaviour: the degree to which HCWs’colleagues use nPOC HIV VL monitoring
	Subjective norm	15	The influence of important others on the use of nPOC HIV VL monitoring
	Self-efficacy	16	The degree to which HCWs believe they are able to implement the activities involved in nPOC HIV VL monitoring
	Knowledge	17	The degree to which the HCWs have the knowledge needed to use nPOC HIV VL monitoring
	Awareness	18	The degree to which HCWs have learnt about the content of nPOC HIV VL monitoring
The organization	Formal ratification by management	19	Formal ratification of nPOC HIV VL monitoring by management (e.g., in policy documents)
	Replacement when staff leave	20	The replacement of staff leaving the organisation
	Staff capacity	21	Adequate staffing in the CTC where nPOC HIV VL monitoring is being used
	Financial resources	22	Availability of financial resources needed to use nPOC HIV VL monitoring
	Time available	23	The amount of time available to use nPOC HIV VL monitoring
	Material resources and facilities	24	The presence of materials and other resources or facilities necessary for the use of nPOC HIV VL monitoring as intended
	Coordinator	25	The presence of one or more persons responsible for coordinating the implementation of nPOC HIV VL monitoring in the organisation
	Unsettled organisation	26	The other changes in progress (organisational or otherwise) that represent obstacles to the process of implementing nPOC HIV monitoring
	Information accessible	27	The accessibility of information about the use of the nPOC HIV VL monitoring process
	Performance feedback	28	The feedback to HCWs about their progress with nPOC HIV VL monitoring process
The social, political, and cultural context	Legislation and regulations	29	The degree to which nPOC HIV VL monitoring fits in with existing legislation and regulations established by the competent authorities

HCWs: Healthcare workers, nPOC HIV VL: Near point -of- care HIV viral load.

### Data collection procedures

Before starting the interviews, we provided all HCWs with a thorough explanation of the study, including its objectives and procedures, and obtained written informed consent. The interviews were conducted by trained researchers with backgrounds in qualitative research, comprising PM (MSW, PhD candidate), AM (MPH), RM (MPH), and RA (MD). All Tanzanian health researchers are familiar with HIV service contexts. All interviewers had prior experience working with HCWs in HIV care settings and none had a prior relationship with the participants before recruitment. Researcher reflexivity was considered throughout the study. PM, with a background in social science and PhD candidate, played a role in coordinating data collection. PM and other research assistants conducted clinic observations and in-depth interviews with HCWs at the selected sites. Then, PM led regular debriefing sessions with the research assistants for emerging themes and ensured the use of neutral probing language. HCWs were interviewed at three time points (months 0, 1 and 6).

Saturation was assessed by continuously reviewing the data throughout the interview process to determine whether new themes were emerging. While saturation was reached across most of the MIDI domains, the themes varied slightly by time point: baseline interviews focused more on provider knowledge, self-efficacy and compatibility with existing practices, while post-implementation interviews introduced new perspectives on organisational coordination, workflow, and client responses to nPOC HIV VL testing. At month six of the interviews, no new themes emerged, indicating that full saturation had been achieved across sites and domains.

#### Data collection tools.

We conducted in-depth interviews (IDIs) lasting 30–45 minutes, using a semi-structured interview guide we initially developed based on the MIDI questionnaire by Fleuren et al., which identifies key factors influencing the implementation of an innovation [17]. The guide was pilot tested by the research assistants to confirm its clarity and reliability before data collection began. The guide evolved across time points: baseline questions focused on prior knowledge, existing practices, and anticipated barriers; post-implementation prompts (months 1 and 6) were added by the research team (PM, AM, MSB) following team debriefings. These adaptations allowed to reflect emerging themes from early interviews and explore observed workflow changes, client responses, and sustainability concerns. To ensure the relevance of MIDI to the Tanzanian HIV service delivery context, the research team discussed and developed questions and prompts consistent with local terminology, health system structures, and HIV care practices in Tanzania. No domains were excluded or merged; rather, the language and examples used in the interview tools were contextualised to ensure HCWs could easily understand them. For example, constructs related to financial and material resources were expanded to capture recurrent supply chain constraints, electricity reliability, and infrastructure limitations frequently observed in Tanzanian clinics. However, descriptive and subjective norms proved difficult to operationalise in the Tanzanian context, hence the results were not informative and are not reported.

Data were also collected through structured observations in the clinics before and after nPOC HIV VL monitoring implementation using predefined checklists to systematically assess workflow, adherence to procedures, and consistency across sites. Informal observations were conducted concurrently to capture natural interactions, workflow adaptations, and contextual factors that might not be captured in the structured checklists. The final versions of all observation checklists and interview guides are provided in S2 Appendix. The observation and interview tools were developed in English and later translated into Kiswahili. Research assistants did back-translation and tested the tools before data collection to confirm their clarity and reliability. During clinic observations and interviews with HCWs, trained research assistants took detailed written notes. We audio-recorded, transcribed verbatim, and then translated the interviews into English. The transcripts were not returned to participants for review or comment and all coding and thematic analysis were conducted directly by the research team.

### Data analysis

We conducted a deductive thematic framework analysis using the MIDI tool, with an inductive approach added. All transcripts were imported into NVivo 12 for coding and organisation. Two researchers (PM and AM) double coded the first six transcripts using a preliminary codebook based on the MIDI framework. During this phase, inductive codes were generated to capture data that did not fit within the redefined MIDI categories. Analytic memos were created during this stage and discrepancies were resolved through discussion and consensus leading to refinement of the coding framework. PM applied the refined combined deductive (MIDI-based) codes with inductively derived subthemes to the remaining 69 transcripts. For consistency, we repeatedly reread the transcripts during analysis and held ongoing discussions within the research team to confirm interpretation. We used NVivo 12 to organise the codes into nodes, highlighting them with coding stripes for consistency, and to track analytic decisions via memos, making the process clear and easy to follow. We created a summary table linking codes to quotes across the four MIDI domains to clearly show how the data aligned with the framework. Credibility was enhanced through: (1) triangulation of IDIs with structured clinic observations; (2) peer debriefing with co-authors to make sure our interpretation makes sense; and (3) use of the MIDI framework as a guide to organise and check our coding of the data. All participants shared similar experiences, and no conflicting or contradictory responses were identified during analysis. The full coding tree and a quote code matrix are provided as supplementary files, S3 Fig and S4 File, respectively.

### Ethical clearance

The study obtained ethical approval with the certificate NIMR/HQ/R8.a/Vol. IX/4140 from the College Research and Ethics Review Committee at Kilimanjaro Christian Medical University College, as well as the National Health Research Ethics Committee of the National Institute for Medical Research. Written informed consent was obtained from each study participant prior to enrolment. Participant information sheets and informed consent forms were translated from English into the Swahili language most commonly used in Tanzania. Each participant (or parent/guardian, where applicable) signed two copies of the informed consent form: one copy was retained by the participant and the other by the research team for documentation. For individuals unable to read or write, consent was provided using a thumbprint, witnessed by an impartial witness chosen by the participant. For participants under 18 years who were not mature or emancipated minors, parent or guardian consent was obtained in addition to the child’s assent (for those aged 8–17 years). Mature or emancipated minors, such as married adolescents, parents, or those heading households, were permitted to provide their own consent, though they were encouraged to involve parents or guardians if safe to do so. This study is reported in accordance with Consolidated Criteria for Reporting Qualitative Research (COREQ) guidelines provided as S5 Checklist.

## Results

### Overview of results and analytical framework

The results are presented in alignment with the study objectives: (1) to investigate factors that contributed to the successful implementation of nPOC HIV VL monitoring and (2) to explore challenges faced by HCWs during implementation. The implementation process of nPOC HIV VL monitoring was guided by the MIDI framework, with findings organised into its four main themes. Within each theme, subthemes were identified based on the 29 MIDI subdomains. To provide context for the findings, we first describe the characteristics of participants and the pre-implementation context. Finally, we describe the key facilitators and challenges in two sections, each organised by the MIDI domains. All quotations are identified by participant cadre, gender and age (e.g., Nurse, Female, 34 years). While most participants’ accounts aligned with major themes, some highlighted challenges they encountered, reflecting variations in perspectives.

### Participant characteristics

A total of 75 interviews were conducted among 43 HCWs across three time points using a design of longitudinal follow up and cross-sectional sampling. At baseline (T0), 33 HCWs participated in interviews. At one month (T1), 25 participants were interviewed, this included 19 participants returning from T0 and 6 new participants. Of the 33 participants at T0, 14 did not attend T1, of whom 7 later returned at T2. Among the 25 participants interviewed at T1, 8 participants were unavailable for further follow-up due to staff transfers, annual leave, or competing clinical duties. By the sixth month of follow-up (T2), 17 participants were interviewed, comprising 6 participants returning from T1, 4 new participants, and 7 participants from T0 (see S1 Table).

Most participants at baseline were female (82%) and most comprised individuals aged 30–39 years (37%). Nurses represented the largest professional group at baseline (49%). However, at six months, the participant profile shifted, with a higher representation of doctors (53%) and a more balanced gender distribution (53% female). The socio-demographic characteristics for each time point are detailed in Table 2 below.

**Table 2:** Demographic characteristics of healthcare workers: Before, one month and six months after nPOC HIV VL monitoring implementation.

Variable	Category	Before nPOC (n = 33)	One-month nPOC (n = 25)	Six months nPOC (n = 17)
Age range	20-29	8 (24%)	9 (36%)	6 (35%)
	30-39	12 (37%)	6 (24%)	6 (35%)
	40-49	10 (30%)	5 (20%)	1 (6%)
	50-59	3 (9%)	5 (20%)	4 (24%)
Gender	Male	6 (18%)	9 (36%)	8 (47%)
	Female	27 (82%)	16 (64%)	9 (53%)
Occupation	Nurses	16 (49%)	6 (24%)	5 (29%)
	Doctors	8 (24%)	7 (28%)	9 (53%)
	Lab technicians	4 (12%)	5 (20%)	0 (0%)
	CHWs	3 (9%)	2 (8%)	1 (6%)
	Data clerks	2 (6%)	5 (20%)	2 (12%)

nPOC = Near point of care, CHWs = Community healthcare workers.

### Pre-implementation context

Before the implementation of nPOC HIV VL monitoring, we conducted observations at the clinic and interviews with HCWs on the standard operating procedures (SOPs). Findings confirmed the standard operations as described in the Tanzania national HIV VL monitoring guidelines. HCWs explained to the client why the VL test was being conducted. Blood was drawn and sent to the central laboratory for processing and testing. The results usually took two weeks to one month to return to the clinics and the HCWs would inform the client of the results during a follow-up visit. Clients with a suppressed viral load (VL) were encouraged to continue taking their medication as prescribed. Those with high VL levels received counselling and were enrolled in more intensive adherence support programs.

### Factors contributing to successful implementation of nPOC HIV VL monitoring

In all four MIDI domains – the intervention, the provider, the organisation, and the socio-political context – HCWs mentioned factors that contributed to the successful implementation of nPOC HIV VL monitoring.

Regarding determinants associated with the intervention, the introduction of nPOC HIV VL monitoring gave HCWs a new responsibility: for clients whose VL was high (≥ 1000 copies/mL), the HCWs needed to clearly explain the new testing process, before taking a new sample for an nPOC test for confirmation, the results of which would be available within approximately two hours. If the VL was high, this allowed for same-day targeted counselling to address barriers to adherence and decide about the next steps (procedural clarity). HCWs trusted the nPOC HIV VL test results, considering them reliable for assessing client treatment progress and VL status. A doctor had this to share:

*“We interpret the results correctly by understanding whether a client’s viral load is suppressed or high, which helps us assess treatment progress: a suppressed viral load indicates effective treatment, while a high viral load signals the need for enhanced adherence counselling and further monitoring.” (Doctor, Male, 28 years)* (correctness)

Complete information on the procedures and steps of nPOC HIV VL monitoring was available through the SOPs, which ensured quality and continuity of care. The SOPs supported the HCWs in adhering to professionally accepted standards when providing nPOC HIV VL monitoring services. A lab technician reported that:

*“We cover everything according to the standard operating procedures*; *from sample collection and methods, we use to ensure accurate results. There are many aspects to consider.” (Lab technician, Male, 37 years)* (completeness)

HCWs highlighted that nPOC HIV VL monitoring aligned with the values and working methods in place and that the same-day results made it easier to observe client outcomes and take timely actions. A doctor reported that:

*“Near POC HIV VL monitoring is the same as the existing viral load monitoring system in terms of procedures; the only difference is that with POC, the results come back in a short time.” (Doctor, female, 24 years*) (compatibility)

The HCWs mentioned that receiving their clients’ results on the same day instead of waiting weeks was helpful for them. It enabled them to understand the clients’ condition more quickly and decide on the next steps and has made it easier to comprehend the clients’ progress more thoroughly. A doctor noted as:

*“When the client’s results are ready, I receive them from the laboratory. The same day results make it easier to know the client’s results and undertake the necessary management.” (Doctor, female, 24 years)* (Observability)

Regarding the provider, the interviews with HCWs noted that the implementation of nPOC HIV VL monitoring brought several benefits. HCWs highlighted that, instead of transporting the sample to the central laboratory, they could now collect and test samples at the nearby lab using nPOC machine, which made them feel more in control of the process. A doctor reported:

*“Our regular procedure takes much longer to get results compared to near point of care VL monitoring, where results come on the same day, which in turn helps us to do early counselling.” (Doctor, female, 24 years)* (personal benefits)

Where possible, HCWs included an explanation of what a high or low VL meant as part of their health counselling to promote treatment adherence. The timely delivery of results, along with accurate visibility of low or high VL, was seen as motivating to the clients and rewarding to the HCWs, as it realised the fruit of their work. A nurse highlighted that:

*“It is advantageous for the client to receive their results on the same day. This allows them to understand their next steps and receive any necessary counselling.” (Nurse, female, 35 years)* (*outcome* expectations)

HCW felt that the implementation of nPOC HIV VL monitoring not only improved their efficiency but also gave them a chance to do what they feel responsible for providing accurate results to their clients during same-day targeted counselling. A doctor reported:

*“When you see a client has a high viral load, you cannot be happy, because your work requires you to make sure the person has received the POC service and is suppressed. So, you must start counselling and give him new education immediately…” (Doctor, male, 42 years) (professional obligation)*

Strong teamwork was key within the organisation. HCWs described how they supported each other by dividing their tasks; if one took a sample, another would process it and the third might provide counselling. A nurse explained:

*“In our clinic, we work together as a team. If I am occupied with another client, a colleague will step in to collect the sample or complete the forms. This kind of support makes it possible to keep the service running smoothly even on busy days.” (Nurse, female, 35years)* (social support)

Observation at the clinic confirmed that HCWs helped each other, which made things easier in every procedure of nPOC HIV VL monitoring.

HCWs expressed confidence in their ability to effectively conduct nPOC HIV VL monitoring. With the recent advancements and improved protocols, they are now well-equipped to carry out the nPOC HIV VL monitoring process accurately and efficiently. It was noted that:

*“In the beginning, we did not understand the POC processes very well. But after receiving the training and working with the system over time, we have gained more confidence and now we understand how to manage the procedures better.” (Doctor, female, 42 years)* (self-efficacy)

Having the understanding required to effectively perform nPOC HIV VL monitoring was noted by HCWs. This included familiarity with the technology, comprehension of its features and the ability to apply relevant skills to make full use of the innovations. A doctor highlighted:

*“I know how the viral load test works, the importance of collecting a good sample and how to store and transport it to ensure accurate results.” (Doctor, female, 58 years)* (knowledge)

In general, HCWs were eager to learn more about nPOC HIV VL monitoring and a few of them mentioned requiring refresher training.

HCWs felt they carried a big responsibility for the implementation of nPOC HIV VL monitoring. They reported that they initially lacked awareness of nPOC HIV VL monitoring, but that the training they had received helped them understand the contents, follow the procedures and interpret the results. A lab technician confirmed this:

*“Before implementation of POC, we had no idea about doing viral load tests ourselves. We used to send everything to [the]central laboratory. It was new for us.” (Lab technician, female, 36 years)* (awareness)

Regarding determinants associated with the organisation, after the HCWs received training on nPOC HIV VL monitoring, they reported receiving formal commitment by the management of the organisation, enabling employees to implement the intervention well. A nurse confirmed that:

*“…the management supported us in implementing the point-of-care viral load testing. They allowed us to adjust duties so that someone is available to run the nPOC test machine and attend to clients. Without that support from management, it would have been difficult to integrate it into our daily routine.” (Nurse, female, 34 years)* (formal ratification by management)

HCWs had dedicated time to incorporate nPOC HIV VL activities into their daily schedules, as observed during clinic observations and informal conversations. This helped ensure that the nPOC HIV VL monitoring service did not interfere with other clinic activities, such as HIV care and treatment services (time available).

HCWs noted that it was easy to find information regarding the intended procedures for conducting nPOC HIV VL monitoring at the facility. The accessible information included detailed guidelines that outlined best practices and effective implementation strategies. Access to information ensured HCWs had a clear understanding of how to apply the innovation effectively. A nurse highlighted that:

*“When we need to check guidelines or confirm the correct procedure for viral load testing, the information is available in the clinic in printed form. This makes it easy to refresh our memory and follow the correct steps without delay.” (Nurse, female, 34 years)* (information accessibility)

The organisational leadership showed a significant contribution to the implementation of nPOC HIV VL monitoring by collaborating well with staff and providing regular performance feedback on the progress of nPOC HIV VL monitoring. The feedback was frequent and aimed to show progress in the implementation of nPOC HIV VL monitoring, including the delivery of nPOC results and improvements in treatment adherence counselling. A lab staff member noted:

“*When we submit nPOC viral load testing reports, the management and sometimes the national program give us feedback on our performance. This helps us understand where we are doing well and where we need to improve.” (Lab technician, male, 38 years)* (performance feedback)

Certain HCWs commented positively on the socio-political context of the intervention. They viewed the implementation of GeneXpert as a most welcome inclusion, in line with best international practice as promoted by the WHO, even though national guidelines for practice had yet to accept such innovation. From a policy perspective, a nurse argued:

*“I don’t think the POC test violated any of the Ministry’s guidelines. This is because the viral load test is done in the same way as it’s usually done. The only difference is that with point-of-care, we get the results on the same day. While if we follow the entire standard procedure [of centralised testing], we end up delaying the client. I think this method is very good.” (Nurse, female, 49 years)* (legislation and regulation)

### Challenges in implementing nPOC HIV VL monitoring

Despite the positive intentions, several challenges arose and affected implementation. Regarding the innovation, at the first stage of the intervention, HCWs encountered difficulties using the GeneXpert machine. The machine requires precise amounts of blood and if there is too little blood, it causes an error and gives the wrong results (complexity).

HCWs also raised the challenge that they face in delivering timely results to clients. To address this, they adapted their service delivery by offering flexible options to accommodate clients’ schedules and minimise inconveniences. A nurse noted that:

*“Because of delays and clients having other commitments, we give them the option to go home and then we call them when their results are ready.” (Nurse, female, 42 years)* (relevance for client)

Regarding determinants associated with the provider, due to too few HCWs available, many had multiple responsibilities, resulting in delays in clinic services and increased pressure on the team. A doctor highlighted:

“*We don’t have enough staff to cover all the services at the same time. Sometimes one person is handling the pharmacy, counselling, and sample collection, which makes it hard to keep up with all the clients.” (Doctor, female, 42 years)* (personal drawback)

Furthermore, through informal conversations with HCWs, they reported that clients were not satisfied with the nPOC test as counselling did not always occur when results were available, particularly when the client had other activities and could not remain on-site to wait for their results. At some participant sites, samples had to be transported to the nearest laboratory by motorbike couriers (boda-boda), which caused delays in obtaining results at three sites Mawenzi, Pasua and Majengo). Based on field observations, transport typically took 20–40 minutes each way, with additional queue time at the receiving laboratory of approximately 30–60 minutes, extending total turnaround time to 3–4 hours or more compared to approximately 90–120 minutes at on site nPOC laboratories (Sinza, Hai). This distinction affected same day counselling feasibility at transport dependent sites (client satisfaction). When this happened, HCWs called clients to share their test results and expected the clients to cooperate further. If the results showed a high VL, clients were expected to return for a separate counselling session (client cooperation).

Regarding determinants associated with the organisation, in some facilities, only a small number of staff were directly involved in nPOC HIV VL monitoring, leading to gaps when a staff member left. Regarding continuity, a doctor reported:

*“If someone trained in POC leaves, we don’t have a structured plan to immediately replace them with someone equally trained. That delays implementation.” (Doctor, female,42years)* (replacement when staff leave)

HCWs highlighted a limited number of staff within their facilities. They emphasised the need for an adequately trained workforce to effectively utilise the nPOC HIV VL monitoring system and ensure its integration into routine clinic operations.

A doctor had this to share:

*“At one centre, there are only a few staff members involved in POC testing. This limited staffing means that if one person leaves or is unavailable, the workload becomes challenging to manage, although the remaining staff try to continue the work.” (Doctor, female, 58 years) (staff capacity)*

The sustainability of the nPOC HIV VL monitoring system was a common concern among HCWs, largely due to limited financial support. HCWs did not identify any financial resources allocated for the long-term implementation of nPOC HIV VL monitoring. A doctor explained:

*“The nPOC HIV VL monitoring equipment is expensive and challenging to obtain; with funding, it is possible to run it. If those resources were not there, we could not afford to maintain [it] ourselves.” (Doctor, Male, 42 years)* (financial resources)

Infrastructure also causes problems; there are often too few service rooms. One doctor highlighted:

*“For my part, I think the rooms are not enough for nPOC implementation. If three or two rooms are present, then the working conditions will be comfortable.” (Doctor, male, 53years) (material resources and facilities)*

In addition, regarding the presence of material resources, a doctor noted that:

*“There are times when we lack gloves and other laboratory supplies, and we have to wait until they are brought from the main hospital.” (Doctor, female, 24years) (material resources and facilities)*

Unreliable electricity and a lack of generators also hindered sample processing. Transportation delays to the nearby laboratory led to sample rejection or result delays. A nurse reported:

*“The biggest challenge is unreliable electricity; we do not have a generator, so sometimes the tests are delayed, or samples get spoiled.” (Nurse, female, 28 years*) (material resources and facilities)

Overcrowded waiting areas and non-client-friendly counselling spaces were observed, which compromised the quality of the service. On the coordination of some of the facilities, a doctor noted that:

“*Sometimes you find the coordination (at the clinic) is not smooth because everyone has their own work. The lab can be busy with other tests, and we are waiting for results. You may have to follow up yourself to make sure clients are not delayed.” (Doctor, female, 42 years) (coordination)*

Furthermore, HCWs reported that the implementation of nPOC HIV VL monitoring took place within an unsettled organisational structure, which added additional coordination needs, requiring regular morning meetings to assign responsibilities. A doctor explained:

*“Roles and responsibilities are not clearly assigned, leading to confusion. Every morning, we have a nPOC HIV VL monitoring meeting, followed by a hospital meeting.” (Doctor, male, 27years)* (unsettled organisation)

Clinic observations revealed several challenges associated with the socio-political context regarding the alignment of nPOC HIV VL monitoring with existing regulations in Tanzania. Consistent with WHO’s 2021 policy, the Tanzania national guidelines used a threshold of >1000 copies/mL to distinguish stable from unstable clients. According to WHO framework, a viral load >1000copies/mL signifies virological failure, while results falling between >50 and **≤** 1000 copies/mL signify low-level viremia, which necessitates enhanced adherence counselling and repeat test after three months. Results **≤** 50 copies/mL are classified as undetectable (target not detected). On top of that, in northern Tanzania, at our study sites, we used 50 copies/mL as imposed by Elizabeth Glaser Pediatric AIDS Foundation (EGPAF). It is important to note that <50 copies/mL represents a laboratory sensitivity benchmark for ‘undetectable’ viral load and is not WHO’S policy threshold for defining suppression (legislation and regulation).

Another challenge involved transitioning from mPIMA to GeneXpert. It was noted that not every POC test can be used for VL monitoring in each setting, depending on the guidelines/practices. GeneXpert nPOC HIV VL monitoring, with its high sensitivity (detection limit approximately 50 copies/mL), was viewed as the best option for providing clinically meaningful results and aligning with future national guideline updates, particularly given the global trend towards more sensitive VL monitoring.

Framed in terms of the determinants of the MIDI framework, HCWs did not experience many problems regarding determinants related to either the intervention or the providers. All subdomains related to the intervention were positively judged, as the procedures for nPOC HIV VL monitoring were clearly described (procedural clarity), based on factually correct knowledge (correctness); the activities described were complete (completeness), aligned with the existing values and working methods in place (compatibility), and the produced outcomes were visible to the user (observability). However, usability of the machine was reduced due to complexity, and relevance to clients was limited by delays in turnaround time for the results.

In terms of provider determinants, most subdomains show enabling factors rather than obstacles: HCWs perceived personal benefits from using nPOC HIV VL monitoring, and believed it helped them achieve their objectives (outcome expectations), aligned with their professional responsibilities (professional obligation), and they expected client’ cooperation. They also reported receiving support from their colleagues (social support), felt confident in their ability to perform the required tasks (self-efficacy), and demonstrated adequate knowledge and awareness of the intervention. Some problems were reported, including personal drawbacks associated with using the system, lower-than-expected client satisfaction, and reduced cooperation. Additionally, expectations of important others (subjective norms) and colleagues’ observed behaviour (descriptive norms) were not clearly mentioned.

At the level of organisational determinants, some subdomains were mentioned as enabling implementation, including formal ratification by management, the time available for nPOC HIV VL monitoring, replacement of staff when they leave, access to information and feedback to HCWs about their use of the nPOC HIV VL monitoring process (performance feedback). However, in the domain of the organisation, many problems were noted, including a lack of replacement when staff left; limited staff capacity, financial and material resources, and facilities, as well as problems with coordination and an unsettled organisation.

On determinants related to the socio-political context, legislation and regulations appeared as a positive subdomain; however, integrating the intervention into national policy proved to be a challenging task.

Overall, the findings show that nPOC HIV VL monitoring improved turnaround time for results and enabled same-day counselling, supported by training, teamwork, and leadership. HCWs adapted well, but faced challenges such as machine errors, staffing shortages, infrastructure issues, management and coordination problems, and a lack of national guideline alignment. Despite these challenges, HCWs remained motivated and viewed the innovation positively.

## Discussion

This qualitative study identified key implementation bottlenecks for nPOC HIV VL monitoring among children and young people in Tanzania from HCWs perspectives, using the MIDI framework. While the intervention itself was accepted by HCWs our findings show that organisational and systemic factors shape the effectiveness of implementation. Below, we discuss these findings in relation to existing literature and their implications for scaling up nPOC HIV VL monitoring in resource-limited settings.

Client satisfaction was mixed, consistent with global evidence. Some studies have found that clients were pleased with nPOC HIV VL testing because the results were available on the same day [18,19], while another study reported many clients were unable to wait for their results and preferred to receive them by phone instead [20]. Similarly, a feasibility study from Uganda highlighted that clients could not wait for their results and recommended establishing fast-track lines for nPOC clients to improve relevance and accessibility [21]. Aligning with these findings, our study show that some clients were not unable to wait for their results, which reduced the perceived benefits of nPOC HIV VL monitoring.

HCWs valued faster turnaround times and same-day counselling, which improved professional satisfaction and client management. The same observation was reported in qualitative studies conducted in South Africa, which reported that HCWs generally found nPOC HIV VL monitoring feasible due to the faster turnaround time of results, enabling efficient client management, but highlighted the need for continuous training to strengthen their knowledge and skills [12,22]. In contrast, one South African study reported significant challenges, particularly heavy workloads, which made it difficult for providers in high-client-volume clinics to fully engage with nPOC technologies [12]. Conversely, other studies have shown that implementing nPOC HIV VL monitoring alongside task shifting can effectively maximise retention in HIV care clinics [18,23]. Similarly, in our study, HCWs emphasised the importance of ongoing training to enhance their knowledge. At the same time, they reported that multitasking and competing responsibilities contributed to delays in delivering nPOC services.

Organisational determinants showed a mixed picture, with strengths coexisting alongside barriers, including staff shortages, insufficient financial and material resources, and high workloads. Similar problems were reported in a rural Tanzanian study, including insufficient staffing, as well as logistical, and infrastructural issues, which caused delays in delivering results [24]. Because samples still had to be transported to the nearest laboratory, this continued to cause delays. This underscores the operational heterogeneity within nPOC implementation: sites with on-site laboratory capacity (Sinza and Hai) achieved turnaround times close to true POC (approximately 90–120 minutes), while sites relying on boda-boda sample transport (Mawenzi, Pasua, Majengo) experienced total delays of 3–4 hours or more, limiting feasibility of same-day counselling and reducing client willingness to wait. Future scale-up planning should explicitly distinguish between these sub-categories of nPOC delivery, as both client communication requirements and system-strengthening priorities differ substantially between them [25]. Similarly, a study by Wang et al. demonstrated that placing POC machines directly at service sites could help mitigate delays caused by transport and waiting times [26]. Insufficient staffing was also highlighted in a study by Engel et al., which concluded that adequate human resources are critical to sustaining nPOC HIV VL monitoring [22].

In our study, HCWs reported that staff shortages and competing responsibilities contributed to delays in providing nPOC services. However, our results go beyond the existing literature by pointing out how organisational challenges not only affected the turnaround time for results but also limited HCWs’ ability to fully integrate nPOC testing into routine workflows. This suggests that, beyond addressing infrastructure and staffing, it may be equally important to strengthen operational systems, and clarify expectations regarding the delivery of results in order to achieve successful implementation.

Finally, in the socio-political context, significant challenges were raised in the legislation and regulation subdomains, such as gaps at the policy level. These findings are consistent with a study by Ganesh et al, which emphasises that without explicit policy authorisations and dedicated funding flows, the implementation of nPOC testing will face difficulties in the transition into sustainable, scaled-up programs [27]. Similarly, our study found that HCWs perceived a lack of clear national policies and limited financial support as key obstacles to integrating nPOC HIV VL monitoring into routine care. However, our results expand on Ganesh et al.’s conclusions by illustrating how these policy and funding gaps directly affect service delivery, leading to delays in adoption and limited implementation of nPOC technologies. It should be noted that according to WHO’s 2021 guideline, a result of low-level viremia (>50 and ≤ 1000 copies/mL) necessitates enhanced adherence counselling and repeat testing. While the < 50 copies/mL value represents the analytical detection limit of platforms such as GeneXpert and is used clinically as a benchmark for ‘undetectable’ viral load, it should not be viewed as a separate policy threshold for failure. Future guideline harmonisation should therefore focus on whether national programmes wish to adopt lower detection benchmarks as a supplementary quality indicator, rather than treating 50 copies/mL as a competing policy standard.

Globally, studies have shown that countries with limited resources face similar challenges in implementing nPOC HIV VL monitoring, including staff shortages, weak policies, and supply problems [11,27,28]. This information confirms that HIV testing and the ability to successfully conduct HIV VL testing and the ability to successfully conduct HIV VL tests are global issues that affect large parts of the world, not just Tanzania.

The overall analysis has shown that there are important interactions between MIDI domains. While the HCWs experienced the intervention simple and compatible with the existing system, they were unable to use it effectively due to a lack of support from their organisations (particularly in terms of workforce and equipment). In addition, the lack of alignment between policy and guidelines (in both the social and political contexts) compounded organisational difficulties, leading to decreased confidence in providers’ abilities to implement the intervention effectively. These connections highlight that successful implementation depends on addressing not only individual and intervention-level determinants but also barriers posed by organisations and the social and political context.

To scale up nPOC HIV VL monitoring, efforts should be made to improve access to HIV VL testing, including training individuals who conduct these tests, improving supply chains, and integrating nPOC services into national laboratory systems. Studies from Malawi and South Africa demonstrate that implementing strong policies, conducting regular staff training, and providing supervisory support enhance both programme sustainability and results [27,29]. Therefore, the scaling up of interventions in Tanzania cannot be viewed solely as an effort to expand access to a technology but must also be seen as part of an overall investment in strengthening the entire health system.

While this study highlights the implementation of nPOC HIV VL monitoring from HCWs’ perspectives, it nevertheless has a few limitations. The main limitation is the study’s dependence on self-reported information from HCWs, which may be biased as they might have overreport positive aspects or underreported the challenges faced in practice. As a result, the findings may overestimate the successes and underestimate the challenges encountered. Although observations at the clinics were conducted to complement the HCWs’ reporting, not all information was captured. Member checking was not conducted because transcripts were not returned to participants for comment, meaning they did not have the opportunity to verify their statements. However, a debriefing session was conducted to mitigate the risk. Furthermore, the data were not in-depth enough to obtain detailed information on the psychological components, such as descriptive and subjective norms. Additionally, the study was conducted in only two regions (Dar es Salaam and Kilimanjaro/Arusha), which are among the most developed regions of Tanzania, and may not fully capture the challenges of nPOC HIV VL monitoring in settings with fewer resources. Despite these limitations, the strengths of the study include the use of the MIDI framework to comprehensively analyse the factors and challenges involved in implementing nPOC HIV viral load monitoring. Additionally, we gathered various perspectives from healthcare workers on pre- and post-implementation of nPOC HIV viral load monitoring over time, providing unique insights into the factors and challenges influencing adoption in the healthcare system.

## Conclusion

Near POC HIV VL monitoring shortens turnaround times and enables immediate counselling. Maximising these benefits requires the prioritisation of investment in staff training, infrastructure, improving sample handling/turnaround time and guideline alignment. Successful implementation of nPOC HIV VL monitoring will enhance the delivery of services and facilitate better outcomes among children and young people living with HIV through timely management and adherence counselling.

### Recommendations

Successful implementation of nPOC HIV VL monitoring in Tanzania requires a multi-faceted approach addressing organisational capacity, policy integration, and system strengthening. While HCWs demonstrated a readiness to adopt nPOC HIV VL technology, sustainable implementation depends on addressing fundamental health system constraints, including staffing, infrastructure, and policy support.

## Supporting information

S1 Table Participant flow across three interview rounds. (DOCX)

S2 Appendix Observation checklists and interview guides. (DOCX)

S3 Fig Code tree. (TIF)

S4 File Code matrix (Excel). (XLSX)

S5 Checklist COREQ checklist. (DOCX)
