# Supplementary material for: Implementation bottlenecks of near point of care HIV viral load monitoring for children and young people in Tanzania: A Qualitative Study
Source: PLoS One. 2026 Jun 12;21(6):e0351304. doi: 10.1371/journal.pone.0351304 (PMC13262835; doi:10.1371/journal.pone.0351304)
Supplement: S1 Table — (DOCX) [file pone.0351304.s001.docx]

| Time point | Total interviewed | Returning participants | New participants |
| --- | --- | --- | --- |
| T0 Baseline | 33 | - | 33 |
| T1(1month) | 25 | 19 fromT0 | 6 |
| T2(6 months) | 17 | 6 from T1;7 from T0 | 4 |
| Total | 75 | - | 43 |

A total of 75 interviews were conducted among 43 healthcare workers (HCWs) across three time points using a mixed longitudinal follow-up and cross-sectional sampling design.
