# Supplementary material for: Implementation bottlenecks of near point of care HIV viral load monitoring for children and young people in Tanzania: A Qualitative Study
Source: PLoS One. 2026 Jun 12;21(6):e0351304. doi: 10.1371/journal.pone.0351304 (PMC13262835; doi:10.1371/journal.pone.0351304)
Supplement: S2 Appendix — (DOCX) [file pone.0351304.s002.docx]

**Observation checklists and Interview guides**

## **HEALTH CARE OBSERVATION (T0)**

*This tool serves to collect observational data in clinics where POC will and has been implemented. The qualitative researcher will, after permission from the head of the clinic/department/unit, situate him/herself in the clinic to make notes based on the below topics. Where possible, researchers should shadow staff so they could for instance stay with the pharmacist or laboratory people or nurse during their activities.*

HEALTH CARE OBSERVATION

Health care observation to be conducted at T0

Location details:

Date:

Time: start……. and finish………….

Instructions:

The observation details can include what you see and what you hear as part of informal conversations with clients and health providers.

I would like you to observe and record all activities taking place at the HIV clinic

###

###

###

Observation topics:

### *When writing down your observations, mention, when relevant, the following topics:*

| Client entry and welcome | Equipment status | Available services |
| --- | --- | --- |
| Availability of guidelines/protocols |  |  |
| Waiting times | Provider practices | Drugs (including shortages) |
| Interaction with providers | Screening | Consumables |
| Costs of services | Adherence monitoring | Health information (posters etc) |
| Interaction with other clients | Health information provided | Staff availability |
|  | Time demands |  |

## **HEALTH CARE PROVIDER INTERVIEW GUIDE BEFORE POINT OF CARE IMPLEMENTATION (T0)**

### Introduction

*Interview guide for interviewing Health care workers who have direct contact with the patient. This will be conducted at T0 after the observations.*

### Instructions

1. *Follow the informed consent procedures*
2. *If consent is given, audio record the interview*
3. *This interview guide is to be used in a flexible manner.*
4. *The aim is to collect in-depth information from the respondent.*

### Introduction to the interview

I want to thank you so much for coming here today and for agreeing to participate in this interview! My name is (…) and I am working on this research project about using the point of care viral load testing that will be implemented in this clinic. You are working in this clinic as (…) and you are involved in processes on viral load testing of people living with HIV. I prepared a few questions and invite you to talk openly and tell me everything that comes to your mind. I expect the interview to last for about half an hour to one hour. It is most helpful when you give me examples from your own work experiences. You can feel free to tell me anything that comes in mind. You have most knowledge about the used intervention, so I really would like to hear from you. All you say remains confidential and the research team will not use your real name when we write about the findings. I would like to record your voice if you give me permission to do that, as it will allow me to concentrate on listening to you, and after we finish our conversation will listen to the recording and write all the things down that we have discussed. If I don’t record, I might miss some important parts of our discussion. The recordings of this interview will be deleted immediately after finalizing this study. However, if you don’t feel comfortable with recording your voice that is fine and I can take notes instead. Do I have your permission to record the interview? Do you have any questions before we start our interview?

**Background information**

I would like to start by finding out a bit about your background

Please tell me a bit about yourself

(probes: age, current role, decision to become health care worker, professional training, career history, present work and day to day activities, previous roles, past training)

| Sex | Female/Male |
| --- | --- |
| Age | ……..years |
| Role in the clinic | ............(MM)/……………(YY) |
| When started working | ............(MM)/……………(YY) |

### Specific Questions

1. **Have you heard about VL monitoring and how have you heard about it?**

Probes: Did you hear something in your clinic? In other places? How was it communicated? To who?

1. **Can you tell me about the activities that you will be asked to do in performing the VL monitoring?**

Probes: Ask about activities such as instructions on what it is, sample taking, counselling, transport, testing/running the machine, preparing the machine/reagent, calling patients

1. **Do you feel the knowledge you have is adequate? If not, what could you be lacking? How can you get to know more?**

### Probes: How/where can you find information about implementing Point of Care viral load testing as intended? What information is available? What is not available? Is it accessible? Will you receive training? Is there somebody you can consult in case you have questions about POC?

*Now let’s talk about your work environment.*

1. **What are the key requirements to implement PoC at your clinic?**

Probes: Are there enough *staff*? Do you have enough *time* to implement the programme as intended in your day-day work? if not how much more time would you need? can you explain more? Is the *equipmen*t sufficient?

### Do you think there might be other issues that can affect the implementation of POC?

### Probes: Understanding of PoC? Willingness of clients to be monitored by PoC? And how do you think they can be mitigated?

### In addition to the implementation of POC, do you expect any other changes in the organisation affecting the workload and work processes in your clinic now or in the foreseeable future

### Probes: (reorganisation, merger, cuts, staffing changes, other innovations)?

### Do you think POC VL monitoring fits in with the existing legislation and regulations?

### Probes: If yes, how? If not, what ways does it not fit?

### Do you have any questions? Are there any issues that you would like to raise?

###

### Thank you for your time and information

##

**HEALTH CARE OBSERVATION (T1 and T2)**

Location details:

Date:

Time: start……. and finish………….

Instructions:

The observation details can include what you see and what you hear as part of informal conversations with clients and health providers.

I would like you to observe and record all activities taking place at the HIV clinic

| Observation checklists |
| --- |
| Client appointment procedures   - How the appointment system work - What takes place when the participant comes for the appointment   What happened at the triage   - Weight check - Blood pressure check - Height check - Nutrition check - Health Education - Other illnesses e.g diabetes and hypertension)   Roles of people at the triage   - Mentor mothers - Adolescents’ peers - Expert clients - Nurses - Nutritionists |
| What happens at each of the delivery service points with the introduction of point of care? What number of staff are included in each delivery service point?   - Clinician - Counselor - Laboratory - Pharmacist - Adolescents center |
| What happens at the different stages of intensive adherence counseling? |
| The process of participants getting the results   - Where do they get the results - When do they get the results - Who gives the results - The interaction taking place when the clinician gives results to the client |
| Scheduling of the next appointment |
| The role of treatment supporters and parents of the children/adolescents |
| Any changes have come with the introduction of PoC test |
| Any costs children/adolescents/parents incurred when waiting for the results?   - Who meets the costs incurred? - How much the patient spends while waiting? - Time lost at work because of waiting for the PoC results |
| Availability of guidelines/SOPs |

Observation details:

**HEALTH CARE PROVIDER INTERVIEW GUIDE AFTER POINT OF CARE IMPLEMENTATION (TI and T2)**

Introduction

Interview guide for interviewing Health care workers who have direct contact with the patient. This will be conducted at T1 and T2 after the observation.

Instructions

1. Follow the informed consent procedures

2. If consent is given, audio record the interview

3. This interview guide is to be used flexibly.

4. The aim is to collect in-depth information from the respondent.

Introduction to the interview

I want to thank you so much for coming here today and for agreeing to participate in this interview! My name is (…) and I am working on this research project about using the point of care viral load testing that will be implemented in this clinic. You are working in this clinic as (…) and are involved in processes on viral load testing of people living with HIV. I prepared a few questions and invited you to talk openly and tell me everything that comes to your mind. I expect the interview to last about half an hour to one hour. It is most helpful when you give me examples from your own work experiences. You can feel free to tell me anything that comes out of your thoughts. You have the most knowledge about the used intervention, so I really would like to hear from you. All you say remains confidential and the research team will not use your real name when we write about the findings. Also, you can refuse any questions and withdraw your participation at any time. I would like to record your voice if you permit me to do that as this is important because after I finish talking to you, I will listen to the recording and write all the things that we have discussed. If I don’t record, I might miss some important parts of our discussion. However, if you don’t feel comfortable with recording your voice that is fine and I can take notes instead. Do I have your permission to record the interview? Do you have any questions before we start our interview?

Background information

I would like to start by finding out a bit about you

Please tell me a bit about yourself (age, decision to become a health care worker, professional training, career history starting with past work then present work and day to day activities)

| Sex | Female/Male |
| --- | --- |
| Age | ……..years |
| Role in the clinic | ............…………… |
| When started working | ............(MM)/……………(YY) |

Specific Questions

1.Could you tell me about the POC viral load monitoring in your clinic?

(MIDI: 1. Procedural clarity, 17 Knowledge, 18 Awareness)

Probes:

How is POC viral load monitoring carried out in your clinic?

What steps do you take when the client has:

(a) unsuppressed viral load

(b)Suppressed viral load?

2. How do you implement the point of care viral load monitoring package?

(MIDI 3. Completeness, 4. Complexity, 5.Compatibility)

Probes: Do you have guidelines (health facility and national guidelines, SOPs) that you

follow to implement POC?

**How do you think point of care approach fits with the working methods at the facility?**

What makes it easier? How?

Why was it easy to implement the POC viral load testing?

**Are you able to implement it well? How?**

3. What difficulties do you find implementing the point of care viral load monitoring package?

Probes: machine difficulties, staffing, infrastructure, client management, administrative

What do you think causes difficulties?

How did you go about those difficulties?

What other ways do you think these difficulties could be made easier?

4. Think back to how you worked before the introduction of the point of care viral load monitoring. How would you compare the previous centralized VL monitoring to the current POC VL monitoring?

(MIDI: 8 personal benefits/drawbacks)

Probes: Does the difference in working affect your ability to work?

If yes, why? How?

How do you find the POC VL monitoring compared to the Centralized viral load

monitoring?

Similarities/differences?

Would you prefer POC to centralized monitoring?

Why would you prefer the POC compared to centralized VL monitoring?

Why not?

5. Now let’s think about the current situation. In what way do you think POC testing is beneficial/not beneficial?

(MIDI: 7 relevance, 9 outcome expectations)

Probes: How is POC VL monitoring helpful to your client?

How is POC beneficial to you or other providers like you? (Probes

job satisfaction, workload)

How did POC affect/ impact your relationship with your client?

What expectations did they have? How was the expectation met?

6. How do you apply your professional skills to the point of care of VL monitoring?

(MIDI: 10 Professional obligation 16 self-efficacy)

Probes: What are the professional difficulties faced?

How did you navigate through difficulties?

Has it changed the way you work? How?

Did you make any adjustments to any of your regular schedules?

Was there a burden? How confident are you using POC?

7. From your observation, what are the client’s perspectives about POC intervention?

(MIDI 11. Client satisfaction, 12 Client cooperation)

Probes: How do they perceive the intervention?

Do they cooperate in the POC viral load monitoring? How?

What do you think makes them cooperate/resist?

How was it received by your client/ respondent?

Have you noticed/ observed a change in their attitude since the introduction of POC?

How acceptable was it by the clients? What do you think made it acceptable?

Was there any resistance? What are they? How did they go about it?

8.In what way do your colleagues assist in the implementation of the POC viral load testing?

(MIDI: 13 Social support, 14 descriptive norm, 15 subjective norm)

Probes: Which steps do they follow?

How adequate is the assistance you receive from them?

What improvements are needed?

How do you feel about the supervision?

If they do not assist you, why do you think they do not assist you?

Do you feel your colleague’s support is important in the implementation of the POC

VL test, if so, how?

Do your colleagues’ actions support you in implementing POC VL, and how?

What kind of assistance with examples? If not, why?

Are there other ways this can be improved?

9. What training did you receive about the POC VL monitoring?

(MIDI: 27 Information accessible)

Probes: From whom, when? What did you learn? Is it helpful?

Was training sufficient? If not, what do you think of refresher training?

Frequency? Examples?

Was the mode of training appropriate?

Is there a need for improvements? If so why and where? If not, why?

10. What arrangement did the management set up in your organisation relating to the use of this POC program?

MIDI: several determinants associated with the organisation (19-26)

Probes: In policy plans, work plans, or other arrangements? E.g., if staff familiar with the POC VL testing leave the organisation? **Staff capacity**

Are there enough financial resources available to implement POC VL testing as

intended? If yes, how?

If not, in what ways does it affect the program?

**Are there available materials and resources to implement PoC program?**

**Is there accessible information about the use of PoC program?**

What other arrangements could be put in place for faster/easier implementation of

the POC program?

What other setbacks did you experience in the arrangement of the use of the POC

program?

11. How do you receive point of care test feedback from participants about progress with the implementation of the program?

MIDI 28 Feedback

Probes: From whom? How often? How is feedback delivered to you? Regularly?

What kind of feedback does the client provide?

How do you respond? When should you receive it?

What other feedback do you need?

**Can you share examples of how point of care has made a difference in a client with the**

**Implementation of the program**

Do participants talk about impact? If yes, what? If not, why?

12. Is the point of care feedback you receive helpful to you and your clients?

(MIDI: 28 Feedback and 6 Observability)

Probes: In what way is it helpful? If not, why is it not helpful? How can it be improved?

**13. What methods do you currently use to deliver point of care test (POCT) results to patients?**

**Probes: Which is the most appropriate method for delivering POC result? The Advantage and**

**disadvantages**

**How do you do it?**

**Are there specific guidelines for delivering POC results?**

**14. What is the typical timeframe for delivering Point of Care Test (POCT) results to patients?**

**Probes: Are there any delays in delivering results? What is the cause of delay?**

**Challenges that may lead to delays in delivering POCT results.**

**How do you handle situations where unexpected delays of results may occur?**

15. Do you have anything else you would like to add? Are there any issues that you would like to raise?

Give an overall summary of the interview and thank the respondent for his/her time
