# Supplementary figures and images for: Implementation bottlenecks of near point of care HIV viral load monitoring for children and young people in Tanzania: A Qualitative Study
Source: PLoS One. 2026 Jun 12;21(6):e0351304. doi: 10.1371/journal.pone.0351304 (PMC13262835; doi:10.1371/journal.pone.0351304)

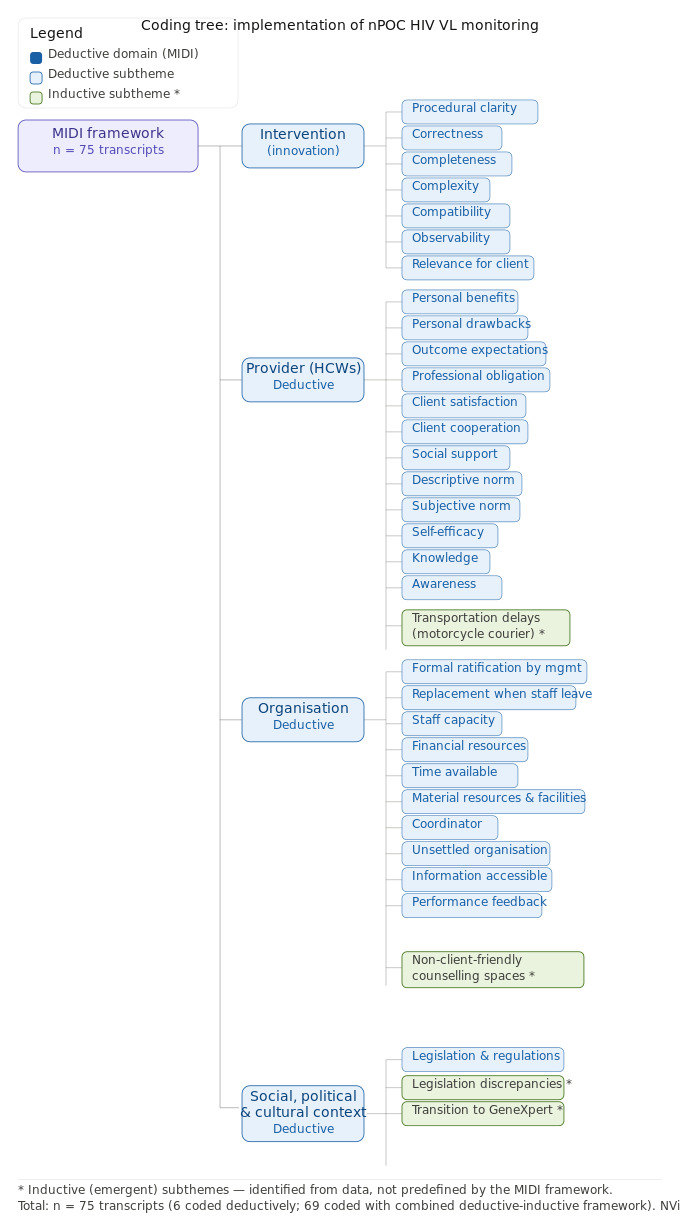

Supplement: S3 Fig — (TIF) [file pone.0351304.s003.tif]
