# Supplementary material for: Implementation bottlenecks of near point of care HIV viral load monitoring for children and young people in Tanzania: A Qualitative Study
Source: PLoS One. 2026 Jun 12;21(6):e0351304. doi: 10.1371/journal.pone.0351304 (PMC13262835; doi:10.1371/journal.pone.0351304)
Supplement: S5 Checklist — (DOCX) [file pone.0351304.s005.docx]

**COREQ (Consolidated criteria for Reporting Qualitative research) Checklist**

| **Topic** | **Item No.** | **Guide Questions/Description** | **Reported on Page No.** | **Notes** |
| --- | --- | --- | --- | --- |
| **Domain 1: Research team and reflexivity** | | |  |  |
| *Personal characteristics* | | |  |  |
| Interviewer/facilitator | 1 | Which author/s conducted the interview or focus group? | N/A | PM, AM, RM, RA |
| Credentials | 2 | What were the researcher’s credentials? E.g. PhD, MD | N/A | PM (MSW)  AM( MPH)  RM(MPH)  RA (MD) |
| Occupation | 3 | What was their occupation at the time of the study? | N/A | Qualitative Research Assistants |
| Gender | 4 | Was the researcher male or female? | N/A | Three females, one male. |
| Experience and training | 5 | What experience or training did the researcher have? | 11 | The interviews were conducted by trained researchers with backgrounds in qualitative research. |
| *Relationship with participants* | | |  |  |
| Relationship established | 6 | Was a relationship established prior to study commencement? | 11 | None of the interviewers knew the participants prior to the study. |
| Participant knowledge of the interviewer | 7 | What did the participants know about the researcher? e.g. personal goals, reasons for doing the research | 11 | Participants did not know the research assistants. |
| Interviewer characteristics | 8 | What characteristics were reported about the interviewer/facilitator? e.g. Bias, assumptions, reasons and interests in the research topic | 11 | All interviewers had prior experience working with HCWs in HIV care settings and none had a prior relationship with the participants before recruitment. Researcher reflexivity was considered throughout the study. PM, with a background in social science and PhD candidate, played a role in coordinating data collection. PM and other research assistants conducted clinic observations and in-depth interviews with HCWs at the selected sites. Then, PM led regular debriefing sessions with the research assistants for emerging themes and ensured the use of neutral probing language. HCWs were interviewed at three time points (months 0, 1 and 6). |
| **Domain 2: Study design** | | |  |  |
| *Theoretical framework* | | |  |  |
| Methodological orientation and Theory | 9 | What methodological orientation was stated to underpin the study? e.g. grounded theory, discourse analysis, ethnography, phenomenology, content analysis | 12 | The interview guide was developed by the measurement determinants of innovation framework (MIDI), and Thematic Analysis was conducted |
| *Participant selection* | | |  |  |
| Sampling | 10 | How were participants selected? e.g. purposive, convenience, consecutive, snowball | 7 | Participants were selected purposively from those involved in implementing nPOC HIV VL monitoring at the intervention sites. |
| Method of approach | 11 | How were participants approached? e.g. face-to-face, telephone, mail, email | 7 | The chief physician of selected intervention health facilities assisted in identifying participants. During the interview day, the participant consented, was interviewed, and was reimbursed. |
| Sample size | 12 | How many participants were in the study? | 16 | 43 Healthcare workers |
| Non-participation | 13 | How many people refused to participate or dropped out? Reasons? | 7 | All HCWs invited agreed to participate and no participants refused or withdrew from the study. However, not all HCWs enrolled at baseline were available for follow up interviews at T1 and T2 due to annual leave, staff transfers and changing clinical responsibilities. |
| *Setting* | | |  |  |
| Setting of data collection | 14 | Where was the data collected? e.g. home, clinic, workplace | 6 | Private office or clinic space of five sites in Tanzania  Moshi – Majengo, Mawezi, Pasua, Hai  Dar es Salaam- Sinza Hospital |
| Presence of non-participants | 15 | Was anyone else present besides the participants and researchers? | N/A | No. No one else was present besides the participants and researchers |
| Description of sample | 16 | What are the important characteristics of the sample? e.g. demographic data, date | 16-17 | A total of 75 interviews were conducted among 43 HCWs across three time points using a mix of longitudinal follow up and cross-sectional sampling design. At baseline (T0), 33 HCWs participated in interviews. At one month (T1), 25 participants were interviewed, including 19 who returned from T0 and 6 new participants. Of the 33 participants at T0, 14 did not attend T1, of whom 7 later returned at T2. Among the 25 participants interviewed at T1, 8 were unavailable for further follow-up due to staff transfers, annual leave or competing clinical duties. By the sixth month of follow-up (T2), 17 participants were interviewed, comprising 6 participants returning from T1, 4 new participants, and 7 who had participated at T0 but not at T1 (see S1 Table).  Most participants at baseline were female (82%) and most comprised individuals aged 30-39 years (37%). Nurses represented the largest professional group at baseline (49%). However, at six months, the participant profile shifted, with a higher representation of doctors (53%) and a more balanced gender distribution (53% female). The socio-demographic characteristics for each time point are detailed in Table 2 below. |
| *Data collection* | | |  |  |
| Interview guide | 17 | Were questions, prompts, guides provided by the authors? Was it pilot tested? | 12 | The guide was pilot tested by the research assistants before data collection to confirm clarity and reliability before data collection began. The guide evolved across time points: baseline questions focused on prior knowledge, existing practices, and anticipated barriers; post-implementation prompts (months 1 and 6) were added by the research team (PM, AM, MSB) following team debriefings |
| Repeat interviews | 18 | Were repeat interviews carried out? If yes, how many? | 16 | A total of 75 interviews were conducted among 43 HCWs across three time points using a mix of longitudinal follow up and cross-sectional sampling design. At baseline (T0), 33 HCWs participated in interviews. At one month (T1), 25 participants were interviewed, including 19 who returned from T0 and 6 new participants. Of the 33 participants at T0, 14 did not attend T1, of whom 7 later returned at T2. Among the 25 participants interviewed at T1, 8 were unavailable for further follow-up due to staff transfers, annual leave or competing clinical duties. By the sixth month of follow-up (T2), 17 participants were interviewed, comprising 6 participants returning from T1, 4 new participants, and 7 who had participated at T0 but not at T1 (see S1 Table). |
| Audio/visual recording | 19 | Did the research use audio or visual recording to collect the data? | 13 | All interviews were audio recorded |
| Field notes | 20 | Were field notes made during and/or after the interview or focus group? | 13 | Field notes were made during the in-depth interview |
| Duration | 21 | What was the duration of the interviews or focus group? | 12 | The average duration of the interviews was 30-45minutes |
| Data saturation | 22 | Was data saturation discussed? | 12 | Yes. Saturation was assessed by continuously reviewing the data throughout the interview process to determine whether new themes were emerging. While saturation was reached across most of the MIDI domains, the themes varied slightly by time point: baseline interviews focused more on provider knowledge, self-efficacy, and compatibility with existing practices, while post-implementation interviews introduced new perspectives on organisational coordination, workflow, and client responses to nPOC HIV VL testing. At month six of the interviews, no new themes emerged, indicating that full saturation had been achieved across sites and domains. |
| Transcripts returned | 23 | Were transcripts returned to participants for comment and/or | 33 | No. Member checking was not conducted because transcripts were not returned to participants for comment, meaning they did not have the opportunity to verify their statements. However, a debriefing session was conducted to mitigate the risk. Furthermore, the data were not in-depth enough to obtain detailed information on the psychological components, such as descriptive and subjective norms |
| **Domain 3: analysis and findings** |  |  |  |  |
| *Data analysis* |  |  |  |  |
| Number of coders | 24 | How many data coders coded the data? | 13 | Two researchers double-coded the data using a combined deductive and inductive approach guided by the MIDI framework. Discrepancies were resolved through discussion and consensus. |
| Description of coding tree | 25 | Did authors provide a description of the coding tree? | 13-14 | Yes, authors explained how the coding structure was developed and organized |
| Derivation of themes | 26 | Were themes identified in advance or derived from the data? | 13 | Yes. We conducted a deductive thematic framework analysis using the MIDI tool, with an inductive approach added |
| Software | 27 | What software, if applicable, was used to manage the data? | 13 | Nvivo 12 and Microsoft Excel for rapid analysis |
| Participant checking | 28 | Did participants provide feedback on the findings? | N/A | No. Participants didn’t provide feedback on the findings |
| *Reporting* |  |  |  |  |
| Quotations presented | 29 | Were participant quotations presented to illustrate the themes/findings? Was each quotation identified? e.g. participant number | 18-27 | Yes, all quotations are identified by participant cadre, gender and age (e.g., Nurse, Female, 34 years). |
| Data and findings consistent | 30 | Was there consistency between the data presented and the findings? | 14 | Yes. Credibility was developed through: (1) triangulation of IDIs with structured clinic observations; (2) peer debriefing with co-authors to make sure our interpretation makes sense; and (3) use of the MIDI framework as a guide to organise and check our coding of the data |
| Clarity of major themes | 31 | Were major themes clearly presented in the findings? | 18-27 | Yes, the major themes were clearly presented according to the four MIDI domains |
| Clarity of minor themes | 32 | Is there a description of diverse cases or a discussion of minor themes? | N/A | No minor themes were identified and the study did not focus on diverse cases |

From: Tong *et al.* *International Journal for Quality in Health Care*; (2007) 19; 6:349-357.
