# Supplementary material for: Implementation bottlenecks of near point of care HIV viral load monitoring for children and young people in Tanzania: A Qualitative Study
Source: PLoS One. 2026 Jun 12;21(6):e0351304. doi: 10.1371/journal.pone.0351304 (PMC13262835; doi:10.1371/journal.pone.0351304)
Supplement: Attachment — Submitted filename: PONE-D-25-65447_reviewer.pdf [file pone.0351304.s006.pdf]

PLOS One

'Clients don't want to wait two hours for their results!' Healthcare workers' Implementation Bottlenecks of Near Point of Care Viral Load Monitoring for Children, Adolescents, and Young People with HIV in Tanzania: A Qualitative Study

--Manuscript Draft--

**Manuscript Number:**	PONE-D-25-65447
**Article Type:**	Research Article
**Full Title:**	'Clients don't want to wait two hours for their results!' Healthcare workers' Implementation Bottlenecks of Near Point of Care Viral Load Monitoring for Children, Adolescents, and Young People with HIV in Tanzania: A Qualitative Study
**Short Title:**	Healthcare workers' challenges for implementing Near Point of Care Viral Load Monitoring for Youth in Tanzania
**Corresponding Author:**	Perry Cyril Msoka, Msc<br>Kilimanjaro Christian Medical Centre<br>Moshi, TANZANIA, UNITED REPUBLIC OF
**Keywords:**	HIV, near point of care viral load, implementation bottlenecks, children adolescents and youth, healthcare workers
**Abstract:**	Background<br>Near point-of-care (n)POC Human Immunodeficiency Virus (HIV) viral load (VL) monitoring, consisting of viral load testing in laboratories close to HIV treatment facilities, improves turnaround time from sampling to result. Other benefits of using nPOC testing are a reduced laboratory workload, and limited loss of results, all leading to improved clinic retention and adherence to treatment. However, the specific implementation bottlenecks are still unclear. This study aims to investigate the bottlenecks in the implementation of nPOC HIV VL monitoring among children, adolescents, and young people (ages 0-24 years) living with HIV, as experienced by healthcare workers (HCWs) in Tanzania.<br><br>Methods<br>We conducted observations in the clinic and in-depth interviews with HCWs from February 2023 to January 2024 at Tanzanian intervention sites of the East Africa Point of Care (EAPOC) Viral Load Monitoring study. The EAPOC study was conducted in four countries in East Africa. This study was a cluster-randomised controlled trial, in which participants were tested at three time points (months 0, 6, and 12). HCWs, involved in the implementation of POC at the intervention sites, were purposively selected for in-depth interviews. We interviewed HCWs before and after the initiation of nPOC HIV VL monitoring. We conducted deductive thematic framework analysis using the Measurement Instrument for Determinants of Innovations (MIDI), to which an inductive approach was added. We used NVivo to organise the data.<br><br>Results<br>Observations and interviews showed that nPOC HIV VL testing improved result turnaround time and enabled same-day counselling, which motivated both HCWs and Clients. Seventy-five healthcare workers participated across three time points (months 0, 6, and 12), which enabled demonstration of gains in knowledge, confidence, and adherence to procedures after training. Near POC, HIV VL was supported by compatibility with existing practices, strong teamwork, and management commitment. However, challenges included clients had to wait at the clinic for their results, the scarcity of resources, such as rooms and electricity, and staff shortages. Finally, delays were observed when nPOC samples had to be transported to nearby laboratories.<br>Conclusion<br>Near POC HIV VL monitoring shortens turnaround times and enables immediate counselling. To maximise these benefits, there is a need to prioritise investment in training staff, infrastructure, improving sample handling/turnaround time and guideline alignment. Developing these will enhance the service delivery and allow for improved outcomes among children, adolescents, and young adults living with HIV.
**Order of Authors:**	Perry Cyril Msoka, Msc

Powered by Editorial Manager® and ProduXion Manager® from Aries Systems Corporation

	Alan Mtenga
	Rehema Maro
	Rhoda Akello
	Iraseni Swai
	Blandina T Mmbaga
	Ria Reis
	Marion Sumari-de Boer
**Additional Information:**	
**Question**	**Response**
**Financial Disclosure**<br><br>Enter a financial disclosure statement that describes the sources of funding for the work included in this submission. Review the submission guidelines for detailed requirements. View published research articles from *PLOS ONE* for specific examples.<br><br>This statement is required for submission and **will appear in the published article** if the submission is accepted. Please make sure it is accurate.<br><br>**Funded studies**<br>Enter a statement with the following details:<br>• Initials of the authors who received each award<br>• Grant numbers awarded to each author<br>• The full name of each funder<br>• URL of each funder website<br>• Did the sponsors or funders play any role in the study design, data collection and analysis, decision to publish, or preparation of the manuscript?<br><br>Did you receive funding for this work?	The author(s) received no specific funding for this work.
**Competing Interests**<br><br>Use the instructions below to enter a competing interest statement for this submission. On behalf of all authors, disclose any competing interests that could be perceived to bias this work—acknowledging all financial support and any other relevant financial or non-	The authors have declared that no competing interests exist

financial competing interests.<br><br>This statement is required for submission and **will appear in the published article** if the submission is accepted. Please make sure it is accurate and that any funding sources listed in your Funding Information later in the submission form are also declared in your Financial Disclosure statement.<br><br>View published research articles from *PLOS ONE* for specific examples.<br><br>**NO authors have competing interests**<br><br>Enter: *The authors have declared that no competing interests exist.*<br><br>**Authors with competing interests**<br><br>Enter competing interest details beginning with this statement:<br><br>*I have read the journal's policy and the authors of this manuscript have the following competing interests: [insert competing interests here]*<br><br>* typeset	
**Ethics Statement**<br><br>Enter an ethics statement for this submission. This statement is required if the study involved:<br><br>• Human participants<br>• Human specimens or tissue<br>• Vertebrate animals or cephalopods<br>• Vertebrate embryos or tissues<br>• Field research<br><br>Write "N/A" if the submission does not require an ethics statement.<br><br>General guidance is provided below. Consult the submission guidelines for	Ethical approvals were obtained from the National Institute for Medical Research (certificate ID NIMR/HQ/R.8c/Vol.I/2749) and the Kilimanjaro Christian Medical University College Research Ethical Committee (certificate ID 2585). All methods and study procedures were carried out in accordance with the Declaration of Helsinki. Participants provided informed consent prior to study entry after the researcher provided a detailed explanation of the study.

Powered by Editorial Manager® and ProduXion Manager® from Aries Systems Corporation

detailed instructions. **Make sure that all information entered here is included in the Methods section of the manuscript.**<br><br>**Format for specific study types**<br><br>**Human Subject Research (involving human participants and/or tissue)**<br>• Give the name of the institutional review board or ethics committee that approved the study<br>• Include the approval number and/or a statement indicating approval of this research<br>• Indicate the form of consent obtained (written/oral) or the reason that consent was not obtained (e.g. the data were analyzed anonymously)<br><br>**Animal Research (involving vertebrate animals, embryos or tissues)**<br>• Provide the name of the Institutional Animal Care and Use Committee (IACUC) or other relevant ethics board that reviewed the study protocol, and indicate whether they approved this research or granted a formal waiver of ethical approval<br>• Include an approval number if one was obtained<br>• If the study involved *non-human primates*, add *additional details* about animal welfare and steps taken to ameliorate suffering<br>• If anesthesia, euthanasia, or any kind of animal sacrifice is part of the study, include briefly which substances and/or methods were applied<br><br>**Field Research**<br><br>Include the following details if this study involves the collection of plant, animal, or other materials from a natural setting:<br>• Field permit number<br>• Name of the institution or relevant body that granted permission	
**Data Availability**<br><br>Authors are required to make all data underlying the findings described fully available, without restriction, and from the time of publication. PLOS allows rare	Yes - all data are fully available without restriction

exceptions to address legal and ethical concerns. See the PLOS Data Policy and FAQ for detailed information.<br><br>A Data Availability Statement describing where the data can be found is required at submission. Your answers to this question constitute the Data Availability Statement and **will be published in the article**, if accepted.<br><br>**Important:** Stating 'data available on request from the author' is not sufficient. If your data are only available upon request, select 'No' for the first question and explain your exceptional situation in the text box.<br><br>Do the authors confirm that all data underlying the findings described in their manuscript are fully available without restriction?	
**Describe where the data may be found in full sentences. If you are copying our sample text, replace any instances of XXX with the appropriate details.**<br><br>• If the data are **held or will be held in a public repository**, include URLs, accession numbers or DOIs. If this information will only be available after acceptance, indicate this by ticking the box below. For example: *All XXX files are available from the XXX database (accession number(s) XXX, XXX.*).<br>• If the data are all contained **within the manuscript and/or Supporting Information files**, enter the following: *All relevant data are within the manuscript and its Supporting Information files.*<br>• If neither of these applies but you are able to provide **details of access elsewhere**, with or without limitations, please do so. For example:<br><br>*Data cannot be shared publicly because of [XXX]. Data are available from the XXX Institutional Data Access / Ethics Committee (contact via XXX) for researchers who meet the criteria for*	All relevant data are within the manuscript and its Supporting Information files.

access to confidential data.<br><br>*The data underlying the results presented in the study are available from (include the name of the third party and contact information or URL).*<br>• This text is appropriate if the data are owned by a third party and authors do not have permission to share the data.<br><br>* typeset	
Additional data availability information:	Tick here if the URLs/accession numbers/DOIs will be available only after acceptance of the manuscript for publication so that we can ensure their inclusion before publication.

Manuscript Click here to access/download;Manuscript;Manuscript.docx

‘Clients don’t want to wait two hours for their results!’ Healthcare workers’ Implementation Bottlenecks of Near Point of Care Viral Load Monitoring for Children, Adolescents, and Young People with HIV in Tanzania: A Qualitative Study

Authors: *Perry Msoka*[1,2,3], *Alan Mtenga*[1], *Rehema Maro*[1], *Rhoda Akello*[4], *Iraseni Ufoo Swai*[1,3,5], *Blandina T. Mmbaga*[1,6, 8], *Ria Reis*[3,7], *Marion Sumari-de Boer*[1,3,6,8]

Affiliations:

1. Kilimanjaro Clinical Research Institute, Moshi, Tanzania
2. Amsterdam Institute for Social Science Research, Amsterdam, the Netherlands
3. Amsterdam Institute for Global Health and Development, Amsterdam, the Netherlands
4. National Institute for Medical Research, Muhimbili
5. Amsterdam UMC, Location AMC, Amsterdam, the Netherlands
6. School of Medicine, KCMC University, Moshi, Tanzania
7. The Children’s Institute, University of Cape Town, Cape Town, South Africa
8. Kilimanjaro Christian Medical Centre, Moshi, Tanzania

Corresponding author: Perry Cyril Msoka
Kilimanjaro Clinical Research Institute
P O Box 2236
Moshi, Tanzania
p.msoka@kcri.ac.tz

Abstract

Background

Near point-of-care (n)POC Human Immunodeficiency Virus (HIV) viral load (VL) monitoring, consisting of viral load testing in laboratories close to HIV treatment facilities, improves turnaround time from sampling to result. Other benefits of using nPOC testing are a reduced laboratory workload, and limited loss of results, all leading to improved clinic retention and adherence to treatment. However, the specific implementation bottlenecks are still unclear. This study aims to investigate the bottlenecks in the implementation of nPOC HIV VL monitoring among children, adolescents, and young people (ages 0-24 years) living with HIV, as experienced by healthcare workers (HCWs) in Tanzania.

Methods

We conducted observations in the clinic and in-depth interviews with HCWs from January 2023 to January 2024 at Tanzanian intervention sites of the East Africa Point of Care (EAPOC) Viral Load Monitoring study. The EAPOC study was conducted in four countries in East Africa. This study was a cluster-randomised controlled trial, in which participants were tested at three time points (months 0, 6, and 12). HCWs, involved in the implementation of POC at the intervention sites, were purposively selected for in-depth interviews. We interviewed HCWs before and after the initiation of nPOC HIV VL monitoring. We conducted deductive thematic framework analysis using the Measurement Instrument for Determinants of Innovations (MIDI), to which an inductive approach was added. We used NVivo to organise the data.

Results

Observations and interviews showed that nPOC HIV VL testing improved result turnaround time and enabled same-day counselling, which motivated both HCWs and Clients. Seventy-five healthcare workers participated across three time points (months 0, 6, and 12), which enabled demonstration of gains in knowledge, confidence, and adherence to procedures after training. Near POC, HIV VL was supported by compatibility with existing practices, strong teamwork, and management commitment. However, challenges included clients had to wait at the clinic for their results, the scarcity of resources, such as rooms and electricity, and staff shortages. Finally, delays were observed when nPOC samples had to be transported to nearby laboratories.

Conclusion

Near POC HIV VL monitoring shortens turnaround times and enables immediate counselling. To maximise these benefits, there is a need to prioritise investment in training staff, infrastructure, improving sample handling/turnaround time and guideline alignment. Developing these will enhance the service delivery and allow for improved outcomes among children, adolescents, and young adults living with HIV.

Keywords: HIV, near point of care viral load, implementation bottlenecks, children, adolescents and youth, healthcare workers

Background

In 2023, UNAIDS reported that 39.9 million people worldwide had HIV, with low and middle-income countries like Tanzania bearing most of the burden.(1) The Tanzanian HIV Impact Survey(2023) stated that adults aged 15 and above had an HIV incidence of 0.18% which interprets to 60,000 new infections yearly.(2) UNAIDS reported that the HIV prevalence among children aged 0 to 14 years in Tanzania reached 0.26% in 2023.(3)

By the year 2030, the UNAIDS goal is to end the HIV/AIDS pandemic by ensuring that 95% of all people living with HIV (PLHIV) are aware of their status, 95% of those diagnosed with HIV infection receive sustained antiretroviral treatment (ART), and 95% of those undergoing treatment achieve viral suppression.(4) Despite significant success in identifying PLHIV (first 95) and initiating these on ART (second 95), challenges still exist in achieving viral load (VL) suppression in PLHIV (third 95), especially among children, adolescents and youth, who mostly depend on caregivers and are affected with higher rates of stigma and psychosocial barriers compared to adults and hence have lower levels of adherence. These influence the rate of lowering the viral suppression and lead to increased vulnerability in this population.(5–7)

In Sub-Saharan Africa, only 72% of PLHIV who are on treatment had a suppressed viral load in 2023, whereas in Tanzania, about 85-88% of those on treatment were virally suppressed. (8) To reach the target of the third 95, the World Health Organisation (WHO) suggests that all individuals receiving ART get a viral load test after 6 months of treatment initiation, followed by another at 12 months and subsequently annually if the individual remains stable on ART. (9) Viral load testing stands out as the most reliable method for monitoring treatment failure in ART. However, the existing standard approach of centralised VL testing, conducted at specialised laboratories, faces several challenges, including delayed results due to transportation issues, sample mislabelling, and the misplacement of results. (10) These challenges highlight the need for innovative strategies such as point-of-care HIV VL monitoring. Point-of-care HIV VL monitoring entails testing at the point where healthcare is provided. The test results can be given on the same day. Also, it can usually be performed by non-laboratory-skilled staff. (9) When testing is done in laboratories close to the treatment facilities, it is called Near Point of Care HIV VL (nPOC HIV VL) testing, which also improves turnaround time from sampling to result.(11) For the implementation of nPOC HIV VL monitoring to be successful, health care workers' (HCWs') perspectives on its implementation are important to take into account in order to improve the feasibility of nPOC testing. One study showed that healthcare workers, who are highly trained and already have

many responsibilities, find that conducting nPOC HIV VL testing adds to their burden, as these HCWs now have to do extra tasks like sampling, running a machine and also directly conduct counselling. (12) A qualitative study carried out in Southwestern Uganda explored HCWs' perceptions of POC HIV VL monitoring's ability to provide faster and more convenient diagnostic results for the clinical management of HIV-positive clients and identified challenges to POC HIV VL monitoring, including a lack of training for HCWs and limited test accuracy. (13) In addition, a study from Kenya reported some challenges in implementing nPOC HIV VL monitoring, which included a shortage of human resources, added burden to the routine workload, power outages, and unreliable electricity. (14)

Since nPOC HIV VL is a new recommended way of delivering fast and efficient VL testing, there is limited evidence regarding the specific implementation bottlenecks of nPOC HIV VL monitoring in Tanzania. Therefore, our qualitative study focused on exploring the experience of healthcare workers regarding implementation bottlenecks of nPOC HIV VL monitoring among children, adolescents, and young people with HIV in Tanzania. The specific objectives were (1) to investigate factors that contribute to good implementation of nPOC HIV VL monitoring and (2) to evaluate challenges faced by HCWs in implementing nPOC HIV VL monitoring.

Methodology

Study design and population

We conducted an explorative qualitative study from January 2023 to January 2024 involving laboratory technicians, nurses, doctors, clinical officers, data clerks, pharmacists, peer educators, and counsellors. The participant's recruitment period began on 11th February 2023 and ended on 28th February 2024. Data were collected through semi-structured, in-depth interviews with healthcare workers (HCWs) who had direct contact with clients in care and treatment centres (CTCs), as well as semi-structured observations.

Study area and setting

We conducted this study in the Kilimanjaro region in the Northern part of Tanzania and the Dar es Salaam region on the East coast of Tanzania. The data collection was part of the EAPOC study, which was a cluster-randomised controlled trial conducted in four countries in East Africa. Clusters, consisting of one or more HIV treatment centres, were randomised into the intervention arm using nPOC HIV VL monitoring or in the control arm using conventional centralised testing for viral load. The study evaluated the effect of nPOC HIV VL monitoring compared to standard centralized monitoring on viral load suppression by taking blood samples at months 6 and 12 of follow-up. We conducted observations in the clinic and in-depth interviews with HCWs at intervention sites of the East Africa Point of Care (EAPOC) study.

In Tanzania, the study was conducted at five Care and Treatment Centres, including a Referral Hospital (Mawenzi in Kilimanjaro), two district hospitals (Sinza in Dar es Salaam and Hai in Kilimanjaro), and two health centres (Pasua and Majengo, both in Kilimanjaro). For Sinza and Hai sites, nPOC HIV VL tests were performed in the hospital laboratories located close to the same clinics. Samples from the Mawenzi, Pasua, and Majengo sites were transported by bodaboda (motorcycle couriers) to the Kilimanjaro Clinical Research Laboratory for testing. All testing was conducted using GeneXpert machines, which can process up to four samples at a time and provide results in approximately 90 minutes.

Sampling of participants

The HCWs to be interviewed were selected purposively from those involved in implementing nPOC HIV VL monitoring at the intervention sites, that is, healthcare workers (1) attending PLWHIV at Care and Treatment Centres (CTC) in one of the five selected health facilities, (2) who are involved in viral

load monitoring, (3) who were part of EAPOC (4) who were providing healthcare treatment and advice based on formal training and experience, such as doctors, nurses, and laboratory technicians and (5) who were not formally trained in providing healthcare, like data managers, peer educators and counsellors. We aimed for maximum variation, including a broad range of professional cadre involved in implementing nPOC HIV VL monitoring. The chief physician of selected intervention health facilities assisted in identifying locally practising HCWs who are currently or were previously involved in HIV client diagnostics and management and staff involved in the EAPOC study. We explained the study thoroughly to the healthcare workers. We then asked for written informed consent.

Theoretical framework

To investigate the implementation bottlenecks, we focused on the feasibility of nPOC HIV VL monitoring, and we used the Measuring Instrument for Determinants of Innovations (MIDI) theoretical framework (13). The MIDI framework is designed to help implementation researchers improve their understanding of the critical determinants that may affect the implementation of an intervention in order to better target the implementation strategy. The framework consists of 29 subdomain constructs divided into four groups of determinants (domains) associated with (1) the intervention, (2) the provider, (3) the implementing organisation, and (4) the social, political, and cultural context. We operationalised these subdomains for the intervention in the nPOC HIV VL monitoring study, as presented in Table 1.

Table 1: Items and constructs of the adapted MIDI for near point of care HIV VL monitoring

Domain	Subdomain	Operationalisation
The Intervention	1. Procedural Clarity	The extent to which nPOC HIV VL monitoring is described in clear steps/procedures
	2. Correctness	The degree to which nPOC HIV VL monitoring is based on factual, correct knowledge
	3. Completeness	The degree to which the activities described in nPOC HIV VL monitoring are complete
	4. Complexity	The degree to which nPOC HIV VL monitoring is complex
	5. Compatibility	The degree to which nPOC HIV VL is compatible with the values and working methods in place
	6. Observability	The visibility of the outcomes for the user, for example, whether the outcomes of the laboratory test are clear to the user
	7. Relevance for the client	The degree to which the user believes nPOC HIV VL monitoring is relevant for his/her client
The provider	8a. Personal benefits	The degree to which using nPOC HIV VL has advantages for the HCWs themselves

	8b. Personal drawback	The degree to which using nPOC HIV VL has disadvantages for the HCWs themselves
	9. Outcome expectations	The perceived probability and importance of achieving the HCW objectives as intended by nPOC HIV VL monitoring
	10. Professional obligation	The degree to which nPOC HIV VL monitoring fits in with the tasks for which the HCW feels responsible when doing his/her work
	11. Client satisfaction	The degree to which the HCW expects Clients to be satisfied with nPOC HIV VL monitoring
	12. Client cooperation	The degree to which the HCW expects Clients to cooperate with (n) POC HIV VL monitoring
	13. Social Support	The support experienced or expected by the user from important social referents relating to the use of nPOC HIV VL monitoring
	14. Descriptive norm	Colleagues' observed behaviour: the degree to which colleagues use nPOC HIV VL monitoring
	15. Subjective norm	The influence of important others on the use of nPOC HIV VL monitoring
	16. Self-efficacy	The degree to which the HCW believes he or she is able to

		implement the activities involved in nPOC HIV VL monitoring
	17. Knowledge	The degree to which the HCW has the knowledge needed to use nPOC HIV VL monitoring
	18. Awareness	The degree to which the HCW has learnt about the content of nPOC HIV VL monitoring
The organization	19. Formal ratification by management	Formal ratification of nPOC HIV VL monitoring by management (e.g. in policy documents)
	20. Replacement when staff leave	The replacement of staff leaving the organisation
	21. Staff Capacity	Adequate staffing in the CTC where the nPOC HIV VL monitoring is being used
	22. Financial resources	Availability of financial resources needed to use nPOC HIV VL monitoring
	23. Time available	The amount of time available to use nPOC HIV VL monitoring
	24. Material resources and facilities	The presence of materials and other resources or facilities necessary for the use of nPOC HIV VL monitoring as intended
	25. Coordinator	The presence of one or more persons responsible for coordinating the implementation of nPOC HIV VL monitoring in the organisation

	26. Unsettled organisation	The other changes in progress (organisational or otherwise) that represent obstacles to the process of implementing nPOC HIV monitoring
	27. Information Accessible	The accessibility of information about the use of nPOC HIV VL monitoring process
	28. Performance feedback	The feedback to the user about progress with the nPOC HIV VL monitoring process
The social, political, and cultural context	29. Legislation and regulations	The degree to which nPOC HIV VL monitoring fits in with existing legislation and regulations established by the competent authorities

HCW: Healthcare worker nPOC HIV VL: Near point of care HIV viral load

Data collection procedures

After providing a thorough explanation of the study, including its objectives and procedures, we obtained written informed consent before we started interviewing. Data collection continued until we reached data saturation. We interviewed HCWs at three time points, before the implementation of nPOC and one month, and six months after implementation. In each site, we intended to interview at least five HCWs at each time point. In each site, we intended to interview at least five HCWs at each time point, reaching a total of sixty interviews. However, we conducted an extra fifteen interviews to reach data saturation.

Data collection tools

We conducted in-depth interviews (IDIs), which lasted 30-45 minutes, using a semi-structured interview guide that we initially developed based on the MIDI questionnaire by Fleuren et al. and further modified during data collection to reflect emerging insights(15). Additionally, we collected data through observation in the clinics using structured observation checklists before and after implementation of nPOC HIV VL monitoring to enable triangulation of data. Informal observations were also conducted during the study. We developed the observation and interview tools in English and later translated them into Kiswahili, since this is the language spoken by people in Tanzania. Research assistants did back translation and tested the tools before data collection to confirm their clarity and reliability. During clinic observations and interviews with healthcare workers, trained research assistants took detailed written notes. We audio-recorded, transcribed verbatim, and then translated the interviews into English.

Data Analysis

We conducted deductive thematic framework analysis using the Measurement Instrument for Determinants of Innovations (MIDI), to which an inductive approach was added. Memos were created by two researchers (PM and AM) for the first six transcripts and then coded with the help of a preliminary codebook that we had made based on the MIDI. PM and AM discussed the results with the last author, MSB. In this process, several new subthemes emerged inductively, which PM and AM proposed to the other authors and added to the framework after agreement was reached. We then applied the combined deductive–inductive coding framework to the remaining sixty-nine transcripts by PM. We used NVivo version 12 Pro for data organisation.

Ethical clearance

We obtained ethical approval with certificate NIMR/HQ/R8.a/Vol. IX/4140 from the College Research and Ethics Review Committee at Kilimanjaro Christian Medical University College, as well as the National Health Research Ethics Committee of the National Institute for Medical Research.

Written informed consent was obtained from each study participant prior to enrolment. Participant information sheets and informed consent forms were translated from English into the swahili language commonly used in Tanzania. Each participant (or parent/guardian, where applicable) signed two copies of the informed consent form. For individuals unable to read or write, consent was provided using a thumbprint, witnessed by an impartial witness chosen by the participant. For participants under 18 years who were not mature or emancipated minors, parent or guardian consent was obtained in addition to the child's assent (for those aged 8–17 years). Mature or emancipated minors, such as married adolescents, parents, or those heading households, were permitted to provide their own consent, though they were encouraged to involve parents or guardians if safe to do so. All consent and assent procedures were reviewed and approved by the National Institute for Medical Research (NIMR) and the Kilimanjaro Christian Medical College Research Ethics and Review Committee (CRERC) prior to implementation. No waiver of consent was granted by the ethics committees.

Results

Socio-demographic characteristics

A total of seventy-five healthcare workers took part in the study at the three data collection time points. At baseline, thirty-three HCWs (44%) were interviewed before POC implementation. After implementation, a total of forty-two HCWs were interviewed during the follow up period. Of these, twenty-five (33%) were interviewed at one month, and seventeen of those participants (at one month) were followed up and interviewed again at six months after implementation, representing 23% of the total sample. Most participants were female, accounting for 52 (69%) of the total. The breakdown of interviewees by profession was as follows: 27 (36%) nurses, 24 (32%) doctors, 9 (12%) lab technicians, 3 (4%) community healthcare workers (CHWs), 9 (12%) data clerks and 3(4%) peer educators.

Table 2: Demographic characteristics of seventy-five healthcare workers, including their division of interviews before, one month and six months of POC implementation, with some HCWs interviewed at more than one time point

Variable	Total (n=75)	Before POC 33(44%)	One-month POC 25(33%)	Six months POC 17(23%)
**Age Range**				
20-29	23(31%)	8(24.2%)	9(36%)	6(35.3%)
30-39	24(32%)	12(36.4%)	6(24%)	6(35.3%)
40-49	16(21%)	10(30.3%)	5(20%)	1(5.9%)
50-59	12(16%)	3(9.1%)	5(20%)	4(23.5%)
**Gender**				
Male	23(31%)	6(18%)	9(36%)	8(47%)
Female	52(69%)	27(82%)	16(64%)	9(53%)
**Occupation**				
Nurses	27(36%)	16(48.5%)	6(24%)	5(29.4%)
Doctors	24(32%)	4(12.1%)	3(12%)	3(17.6%)
Lab technicians	9(12%)	1(3%)	6(24%)	2(11.8%)
CHWs	3(4%)	1(3%)	1(4%)	1(5.9%)
Data Clerk	9(12%)	5(15.2%)	3(12%)	1(5.9%)
Peer educators	3(4%)	2(6.1%)	0(0%)	1(5.9%)

Before the implementation of the nPOC HIV VL monitoring, we conducted observations at the clinic and interviews with HCWs on the standard operating procedures. Findings confirmed the standard operations as described in the Tanzania national HIV VL monitoring guidelines. HCWs explained to the client why the viral load test was conducted. Blood was drawn and sent to the central laboratory for processing and testing. The results usually took two weeks to one month to return. When the results were ready, they would be brought back to the clinics, and HCW would inform the client of the results on a follow-up visit. Clients with suppressed viral load (VL) were encouraged to continue taking their medication as prescribed. Those with high VL levels received counselling and were enrolled in more intensive adherence support programs.

Factors for the good implementation of nPOC HIV VL monitoring

With the introduction of POC HIV VL testing, HCWs obtained a new role: they needed to explain this new testing process to clients clearly for the patient whose VL was high (≥ 100 copies/ml). Then, HCWs would take a new sample for a POC test for confirmation, for which the results would be available within approximately two hours. This allowed for same-day targeted counselling to address barriers to adherence and decide about the next steps if the VL is high.

We conducted further observations at the clinic and interviews with HCWs after implementation of nPOC HIV VL monitoring to assess factors that contribute to good implementation of nPOC HIV VL monitoring and gather their experiences. It was noted that the implementation of nPOC HIV VL monitoring brought several benefits. Healthcare workers highlighted that instead of transporting the sample to the central laboratory, they could now collect and test samples at the nearby lab using nPOC machine which made them feel more in control of the process. A doctor reported:

"Our regular procedure takes much longer to get results compared to point of care, where results come on the same day, so it becomes easier to know the client's results and undertake the necessary management." (Doctor, 24yrs, female, T2), (personal benefits)

Healthcare workers felt that this not only improved their efficiency but also gave a chance to do what they feel responsible for: providing results for their clients on time and same-day targeted counselling, at the right time. A doctor reported:

"When you see a client has a high viral load, you cannot be happy, because your work requires you to make sure the person has received the POC service and is suppressed. So, you must start counselling and give him new education immediately…" (Doctor, 42 yrs, male, T2), (professional obligation)

At the same time, HCW highlighted that nPOC HIV VL monitoring aligns with the values and working methods in place. A doctor reported that:

"Near POC HIV VL monitoring is the same as the existing viral load monitoring system in terms of procedures; the only difference is that with POC, the results come back in a short time." (Doctor, 24 yrs, female, T2), (compatibility)

The information on the use of nPOC HIV VL monitoring was completely available through standard operating procedures (SOPs) used to ensure quality and continuity of care. The SOPs supported the HCWs in adhering to professionally accepted standards when providing nPOC services. A lab technician reported that:

> *"We cover everything according to the standard operating procedures; from sample collection and methods, we use to ensure accurate results. There are many aspects to consider." (Lab technician, 37 years old, Male),* (completeness)

Another doctor had this to share:

> *"We interpret the results correctly by understanding whether a client's viral load is suppressed or high, which helps us assess treatment progress: a suppressed viral load indicates effective treatment, while a high viral load signals the need for enhanced adherence counselling and further monitoring."(Doctor, 28 years old, Male),* (observability)

Healthcare workers felt they carried a big responsibility for the implementation of nPOC HIV VL monitoring. Healthcare workers reported that they initially lacked awareness of the nPOC HIV VL monitoring, but that through the training they received, they learned about the contents of nPOC HIV VL monitoring, which helped them follow procedures and interpret results. A lab technician confirms this:

> *"Before implementation of POC, we had no idea about doing viral load tests ourselves. We used to send everything to central laboratory. It was new for us." (Lab technician, 36 yrs, female T0) (learnt about the content)*

The doctor added that they believed now to be able to conduct the monitoring well, as said by a doctor:

> *"In the beginning, we did not understand the POC processes very well. But after receiving the training and working with the system over time, we have gained more confidence and now we understand how to manage the procedures better." (Doctor, 42 yrs, female T2) (self-efficacy)*

Where possible, HCWs included an explanation of what a high or low viral load meant as part of their health counselling to promote treatment adherence. The timely delivery of results, along with accurate visibility of low or high VL, was seen as motivating to the clients and rewarding to the HCWs, as it realized the fruit of their work. A nurse highlighted that:

> *"It is advantageous for the client to receive their results on the same day. This allows them to understand their next steps and receive any necessary counselling." (Nurse, 35 years old, female, T2)* (outcome expectations)

Further, a nurse had reported:

> *"Even though the process has many stages, I believe I can manage it because I have handled similar testing before, and I know how to follow procedures carefully." (Nurse, 23 yrs, female, T0) (self-efficacy)*

About the issue of knowledge needed to perform nPOC, the doctor highlighted:

> *"I know how the viral load test works, the importance of collecting a good sample and how to store and transport it to ensure accurate results." (Doctor, 58 yrs, female, T1) (knowledge)*

In general, HCWs were eager to learn more about nPOC HIV VL monitoring and a few of them mentioned requiring refresher training.

Strong teamwork was key within the organisation. Healthcare workers described how they supported each other by dividing their tasks; if one took a sample, another would process it and the third might provide counselling. A nurse explained:

> *"In our clinic, we work together as a team. If I am occupied with another patient, a colleague will step in to collect the sample or complete the forms. This kind of support makes it possible to keep the service running smoothly even on busy days." (Nurse, 35yrs, female, T1) (social support)*

Observation at the clinic confirmed that HCWs were helping each other, which makes things easier in every procedure of nPOC HIV VL monitoring.

Healthcare workers reported that the organisational leadership showed a significant contribution to the implementation of the nPOC HIV VL monitoring by collaborating well with staff and providing regular performance feedback on the progress of nPOC HIV VL monitoring. The feedback was frequent and

aimed to show progress in the implementation of nPOC HIV VL monitoring, including the delivery of POC results and improvements in treatment adherence counselling. A lab staff member noted:

> "*When we submit nPOC viral load testing reports, the management and sometimes the national program give us feedback on our performance. This helps us understand where we are doing well and where we need to improve." (lab technician, 38 yrs, male, T2) (performance feedback)*

After the HCWs received training on nPOC HIV VL monitoring, they reported receiving formal commitment by the management of the organisation, enabling employees to implement the intervention well. A nurse confirmed that:

> *"…the management supported us in implementing the point-of-care viral load testing. They allowed us to adjust duties so that someone is available to run the nPOC test machine and attend to clients. Without that support from management, it would have been difficult to integrate it into our daily routine." (Nurse, 34 yrs, female, T2) (formal ratification by management)*

On the political-social ground, certain HCWs viewed the implementation of GeneXpert as a most welcome inclusion, in line with best international practice as promoted by the World Health Organisation (WHO), even though national guidelines for practice had yet to accept such innovation. From a policy perspective, a nurse argued:

> *"I don't think the POC test violated any of the Ministry's guidelines. This is because the viral load test is done in the same way as it's usually done. The only difference is that with point-of-care, we get the results on the same day. While if we follow the entire standard procedure [of*

centralized testing], we end up delaying the client. I think this method is very good." (Nurse, 49yrs, female, T1) (legislation and regulation)

Challenges in implementing nPOC HIV VL monitoring

Despite the good intentions, several challenges arose and affected implementation. Regarding the innovation, at the first stage of the intervention, HCWs encountered difficulties using the GeneXpert machine. This nPOC machine needs precise amounts of blood, and if there is too little blood, it would cause an error and give the wrong results(complexity). Furthermore, through informal conversations with HCWs, they reported that clients were not satisfied with the nPOC test, as counselling did not always occur when results were available, particularly if the client had other activities and could not remain on-site to wait for their results. At some participant sites, samples had to be transported to the nearest laboratory by motorbike couriers (bodaboda), which caused delays in obtaining results. (client satisfaction). When this happened, HCWs expected the client to cooperate with nPOC HIV VL monitoring by calling to share their test results. If the results showed a high viral load, clients were expected to return for a separate counselling session. (client cooperation) Further, HCW added:

"Because of delays and clients having other commitments, we give them the option to go home and then we call them when their results are ready." (Nurse, 42 yrs old, female, T2) (relevance for client)

Additionally, a significant challenge was the shortage of staff. A doctor had this to share:

"At one centre, there are only a few staff members involved in POC testing. This limited staffing means that if one person leaves or is unavailable, the workload becomes challenging to manage, although the remaining staff try to continue the work(Doctor, 58 yrs old, female, T0) (staff capacity)

Healthcare workers had dedicated time to incorporate nPOC HIV VL activities into their daily schedules. (Time availability) This helped ensure that the nPOC HIV VL monitoring service did not interfere with other clinic activities, such as HIV care and treatment services. However, due to too few HCWs available, many had multiple responsibilities, resulting in delays in clinic services and increased pressure on the team. A doctor further highlighted:

> *"We don't have enough staff to cover all the services at the same time. Sometimes one person is handling the pharmacy, counselling, and sample collection, which makes it hard to keep up with all the patients."(Doctor, 42 yrs, female, T2) (personal drawback)*

In some facilities, only a small number of staff were directly involved in nPOC HIV VL monitoring, leading to gaps when any staff member leaves. For continuity, a doctor reported:

> *"If someone trained in POC leaves, we don't have a structured plan to immediately replace them with someone equally trained. That delays implementation." (Doctor, 42yrs female, T2) (replacement when staff leave)*

Further, one nurse emphasized:

> *"If a staff member trained in sample collection leaves, it is important to have someone else trained immediately. Without a replacement, the service stops and clients experience delays in getting tested." (Nurse, 30yrs, female, T0) (replacement when staff leave)*

It was further noted that nPOC HIV VL equipment was expensive and difficult to obtain in the future. (Financial resources) Infrastructure also causes problems; there were often too few service rooms. One doctor highlighted:

> *"For my part, I think the rooms are not enough for nPOC implementation. If three or two rooms are present, then the working conditions will be comfortable."* (Doctor, 53yrs, male, T2) (material resources and facilities)

In addition, when it comes to the presence of material resources, a doctor noted that:

> *"There are times when we lack gloves and other laboratory supplies, and we have to wait until they are brought from the main hospital."* (Doctor, 24yrs, female, T2) (material resources and facilities)

Unreliable electricity and a lack of generators all hindered sample processing. Transportation delays to the nearby lab led to sample rejection or result delays. A nurse reported:

> *"The biggest challenge is unreliable electricity; we do not have a generator, so sometimes the tests are delayed, or samples get spoiled."* (Nurse, 28yrs, female, T2) (material resources and facilities)

Overcrowded waiting areas and non-client-friendly counselling spaces were observed, which compromised the quality of the service. Further, HCWs reported that the implementation of nPOC HIV VL monitoring took place within an unsettled organisational structure, which added additional coordination needs, requiring regular morning meetings to assign responsibilities. A doctor explained:

> *"Roles and responsibilities are not clearly assigned, leading to confusion. Every morning, we have a POC HIV VL monitoring meeting, followed by a hospital meeting."* (Doctor, 27yrs, male, T2) (unsettled organisation)

On the coordination of some of the facilities, a doctor noted that:

Sometimes you find the coordination (at the clinic) is not smooth because everyone has their own work. The lab can be busy with other tests, and we are waiting for results. You may have to follow up yourself to make sure clients are not delayed.''(Doctor, 42yrs, female, T2) (coordination)

Additionally, observation revealed several challenges regarding the alignment of POC VL monitoring with existing regulations in Tanzania. The national guidelines still used a 1000 copies/ml cut-off to distinguish stable and unstable patients, while the new WHO guidelines set the threshold at 50 copies/ml. (legislation and regulation) In our sites, the implementing partners have already implemented this new WHO guideline, which creates a discrepancy between practice and national policy.

Another challenge involves transitioning from mPIMA to GeneXpert. It was noted that not every POC test can be used for VL monitoring in each setting, depending on the guidelines/practices. GeneXpert (n)POC HIV VL monitoring, which aligns with the 50 copies/ml threshold, was the best option for compliance with WHO guidelines and future legislative changes. (legislation and regulation)

Discussion

This qualitative study identified key implementation bottlenecks for near point-of-care HIV viral load monitoring among children, adolescents, and young people in Tanzania from healthcare workers' perspectives. Using the MIDI framework, we found that while healthcare workers accepted the intervention, significant organisational and systemic challenges limit successful implementation.

Overall, the findings show that nPOC HIV VL monitoring improved turnaround time for results and enabled same-day counselling, supported by training, teamwork, and leadership. Healthcare workers adapted well, but faced challenges such as machine errors, staffing shortages, infrastructure issues, management and coordination problems, and a lack of national guideline alignment. Despite these, HCWs remained motivated and viewed the innovation positively.

Framed in terms of the determinants of the MIDI framework, HCWs did not experience many problems regarding determinants related to either the intervention or the providers. All subdomains related to the intervention were positively judged, as the procedures for nPOC HIV VL monitoring were clearly described (procedural clarity), based on factual correct knowledge (correctness); the activities described were complete (completeness), aligned with the existing values and working methods in place (compatibility), and the produced outcomes were visible to the user (observability). However, the relevance to the clients was reduced by the delay in turnaround time for the results.

In terms of provider determinants, most subdomains show enabling factors rather than obstacles: HCWs perceived personal benefits from using nPOC HIV VL monitoring, and believed it helped them achieve their objectives (outcome expectations), aligned with their professional responsibilities (professional obligation), and expected clients' cooperation. They also reported receiving support from their colleagues (social support), felt confident in their ability to perform the required tasks (self-efficacy), and demonstrated adequate knowledge and awareness of the intervention. Some problems were reported,

including personal drawbacks associated with using the system, lower-than-expected client satisfaction, and reduced cooperation. Additionally, expectations of important others (subjective norms) and colleagues' observed behaviour (descriptive norms) were not clearly mentioned.

At the level of organisational determinants, some subdomains were mentioned as enabling the implementation, including formal ratification by management, replacement of staff when they leave, access to information and feedback to the user about the use of the nPOC HIV VL monitoring process (performance feedback). However, in the domain of the organisation, many problems were noted, including shortages of staff, financial resources, material resources, and facilities, as well as problems with coordination and an unsettled organisation. Time availability presented a challenge, as HCWs felt overwhelmed by their workload.

On determinants related to the social, political, and cultural context, legislation and regulations appeared as a positive subdomain; however, integrating the intervention into national policy proved to be a challenging task.

Below, we discuss these findings in relation to existing literature and their implications for scaling up nPOC HIV VL monitoring in resource-limited settings.

Previous studies have reported mixed findings regarding client satisfaction with POC HIV testing. Some studies found that clients were pleased with POC HIV VL testing because the results were available on the same day.(16,17) However, another study reported that many clients were unable to wait for their results and preferred to receive them by phone instead.(18) Similarly, a feasibility study from Uganda highlighted that clients could not wait for their results and recommended establishing fast-track lines for POC Clients to improve relevance and accessibility.(19) Aligning with these findings, our study showed that some of the clients were not able to wait for the results, which reduced the perceived benefits of the

nPOC HIV VL monitoring. Although HCWs were optimistic about the intervention, they suggested that reducing the turnaround time for results could increase client satisfaction and overall effectiveness of the intervention.

The qualitative studies conducted in South Africa reported that HCWs generally found nPOC HIV VL monitoring feasible due to the turnaround time of results being faster, enabling efficient patient management, but highlighted the need for continuous training to strengthen their knowledge and skills.(12,20) In contrast, one SA study reported significant challenges, particularly heavy workloads, which made it difficult for providers in high-patient-volume clinics to fully engage with POC technologies.(12) Conversely, other studies have shown that implementing POC HIV VL monitoring alongside task shifting can effectively maximise retention in HIV care clinics.(21,22) Similarly, in our study, HCWs emphasised the importance of ongoing training to enhance their knowledge. At the same time, they reported that multitasking and competing responsibilities contributed to delays in delivering POC services.

Organisational determinants showed a mixed picture, with strengths coexisting alongside barriers, including staff shortages, insufficient financial and material resources, and high workloads. Similar problems were reported in a rural Tanzanian study, including insufficient staffing, logistical, and infrastructural issues, which caused delays in delivering results.(23) Because samples still had to be transported to the nearest laboratory, this continued to cause delays. This emphasises the difference between point of care and near point of care testing, which needs clear communication about service delivery times and strengthening the systems for utilisation of results.(24) Similarly, a study by Wang et al. demonstrated that placing POC machines directly at the service sites could help mitigate delays caused by transport and waiting times.(25) Insufficient staffing was also highlighted in a study by Engel

et al., which concluded that adequate human resources are critical to sustaining nPOC HIV VL monitoring.(20)

In our study, HCWs reported that staff shortages and competing responsibilities contributed to delays in providing POC services. However, our results go beyond the existing literature by pointing out how the organisational challenges not only affect the turnaround time for results but also limit HCWs' ability to integrate POC testing into routine workflows fully. This suggests that, beyond addressing infrastructure and staffing, strengthening operational systems, and clarifying expectations for delivering results may be equally important for successful implementation.

Finally, in the social and political context, significant challenges were raised in the legislation and regulation subdomains, such as gaps at the policy level. These findings are consistent with the study by Rosen et al, which emphasises that without explicit policy authorisations and dedicated funding flows, implementation of POC testing faces difficulties in transitioning into sustainable, scaled-up programs.(26) Similarly, our study found that HCWs perceived a lack of clear national policies and limited financial support as key obstacles to integrating POC HIV VL monitoring into routine care. However, our results extend Rosen et al.s' conclusions by illustrating how these policy and funding gaps directly affect service delivery, leading to delays in adoption and limited implementation of POC technologies.

Although this study highlights the implementation of nPOC HIV VL monitoring from the HCWs' perspectives, there are a few limitations to our study. Its main limitation is its dependence on self-reported information from HCWs, which may be biased as they might overreport positive aspects or underreport the challenges faced in practice. As a result, the findings can overestimate the success and underestimate the challenges encountered. Although observation at the clinic was conducted to complement the HCWs' report, not all information was captured. Furthermore, the data were not in-

depth enough to obtain detailed information on the psychological components, such as descriptive and subjective norms. Additionally, the study was conducted in only two regions (Dar es Salaam and Kilimanjaro/Arusha), which are among the most developed regions of Tanzania, and may not fully capture the challenges of nPOC HIV VL monitoring in less resource settings. Despite these limitations, the strengths of the study include the use of the MIDI framework to comprehensively analyse the factors and challenges involved in implementing nPOC HIV viral load monitoring. Additionally, we gathered various perspectives from healthcare workers on pre- and post-implementation of nPOC HIV viral load monitoring over time, providing unique insights into the factors and challenges influencing adoption in the healthcare system.

Conclusion

Near POC HIV VL monitoring shortens turnaround times and enables immediate counselling. Maximising these benefits needs prioritisation in investment in training staff, infrastructure, improving sample handling/turnaround time and guideline alignment. Successful implementation of nPOC HIV VL will enhance the delivery of services and facilitate better outcomes among children, adolescents, and young adults living with HIV through timely management and adherence counselling.

Recommendations

A successful implementation of nPOC HIV VL monitoring in Tanzania requires a multi-faceted approach addressing organisational capacity, policy integration, and system strengthening. While healthcare workers demonstrated readiness to adopt nPOC HIV VL technology, sustainable

implementation depends on addressing fundamental health system constraints, including staffing, infrastructure, and policy support.

Ethical Approval

Ethical approvals were obtained from the National Institute for Medical Research (certificate ID NIMR/HQ/R.8c/Vol.I/2749) and the Kilimanjaro Christian Medical University College Research Ethical Committee (certificate ID 2585). All methods and study procedures were carried out in accordance with the Declaration of Helsinki. Participants provided informed consent prior to study entry after the researcher provided a detailed explanation of the study.

Competing interests

The author declares no competing interests

Authors' contributions

PM, AM, MS and RR contributed to the design, research implementation, and manuscript review. PM and AM analysed the results and wrote the manuscript. MS supervised the project. BM, RR and IS reviewed the manuscript. PM, MS and RR contributed to the manuscript writing. AM, RA, RM, and PM contributed to data collection and analysis.

Acknowledgements

We thank all the patients and staff from all the sites participating in the EAPoC-VL study. EAPOC-VL is part of the EDCTP programme supported by the European Union under grant agreement: RIA2019R-2873.The funders had no role in the study design, data collection and analysis, the decision to publish or the preparation of the manuscript. The study is a collaboration between Uganda National Health Research Organisation/Uganda Virus Research Institute (UNHRO/UVRI, Uganda), Karolinska Institutet (Sweden), The Good Samaritan Foundation Kilimanjaro Christian Medical Centre (GSF KCMC, Tanzania), Stichting Amsterdam Institute for Global Health and Development (AIGHD, the Netherlands), London School of Hygiene and Tropical Medicine (MRC/UVRI & LSHTM Uganda Research Unit, Uganda), National Institute for Medical Research

(NIMR, Tanzania), Kenya Medical Research Institute (KEMRI), Center for Global Health Research (CGHR), and the University of Rwanda (UR, Rwanda).

Availability of data and materials

All data generated or analysed during this study can be communicated to the first author upon signing a data transfer agreement in accordance with the nation's policy.

References

1. UNAIDS. Fact sheet 2024 - Latest global and regional HIV statistics on the status of the AIDS epidemic. 2024; Available from: https://www.unaids.org/en
2. Ofisi ya Taifa ya Takwimu. TANZANIA HIV IMPACT SURVEY. Available from: https://www.nbs.go.tz/nbs/takwimu/THIS2022-2023/THIS2022-2023_Summary_Sheet.pdf
3. UNAIDS. 2025 AIDS TARGETS.
4. UNAIDS 2022. Geneva: Joint United Nations Programme on HIV/AIDS;2022.Licence:CC BY-NC-SA 3.0 IGO.
5. Humphrey JM, Genberg BL, Keter A, Musick B, Apondi E, Gardner A, et al. Viral suppression among children and their caregivers living with HIV in western Kenya. 2019; Available from: http://onlinelibrary.wiley.com/doi/10.1002/jia2.25272/full
6. Lebelonyane R, Bachanas P, Block L, Ussery F, Alwano MG, Marukutira T, et al. To achieve 95-95-95 targets we must reach men and youth: High level of knowledge of HIV status, ART coverage, and viral suppression in the Botswana Combination Prevention Project through universal test and treat approach. PLoS One. 2021 Aug 1;16(8 August).
7. Hlophe LD, Tamuzi JL, Shumba CS, Nyasulu PS. Barriers and facilitators to anti-retroviral therapy adherence among adolescents aged 10 to 19 years living with HIV in sub-Saharan Africa: A mixed-methods systematic review and meta-analysis [Internet]. Vol. 18, PLoS ONE. 2023. 1–40 p. Available from: http://dx.doi.org/10.1371/journal.pone.0276411
8. Unaids. People living with HIV — Thematic briefing note — 2024 global AIDS update The

Urgency of Now: AIDS at a Crossroads. 2024;2024(2):1–10. Available from: https://aidsinfo.unaids.org/

9. World Health Organization. Considerations for adoption and use of multidisease testing devices in integrated laboratory networks: information note. Available from: https://iris.who.int/handle/10665/255693

10. UNAIDS. Data 2020. Program HIV/AIDS [Internet]. 2020;1–436. Available from: http://www.unaids.org/sites/default/files/media_asset/20170720_Data_book_2017_en.pdf

11. Tun NN, Smithuis F, Tun NL, Min M, Hlaing MMM, van Olmen J, et al. Near point-of-care HIV viral load testing: Cascade after high viral load in suburban Yangon, Myanmar. PLoS One [Internet]. 2023;18(4 April):1–15. Available from: http://dx.doi.org/10.1371/journal.pone.0272673

12. Msimango L, Gibbs A, Shozi H, Ngobese H, Humphries H, Drain PK, et al. Acceptability of point-of-care viral load testing to facilitate differentiated care: a qualitative assessment of people living with HIV and nurses in South Africa. BMC Health Serv Res. 2020;20(1):1–9.

13. Rasti R, Nanjebe D, Karlström J, Muchunguzi C, Mwanga-Amumpaire J, Gantelius J, et al. Health care workers' perceptions of point-of-care testing in a low-income country - A qualitative study in Southwestern Uganda. PLoS One. 2017;12(7):1–18.

14. Boniface S, Lwilla A, Mahiga H, Pamba D, Geisenberger O, France J, et al. Xpert HIV-1 qual point-of-care testing for HIV early infant diagnosis in Tanzania: experiences and perceptions of health care workers in a 2016 study. AIDS Res Ther. 2024 May 16;21(1).

15. Fleuren MAH, Paulussen TGWM, Dommelen P, Buuren S Van. Towards a measurement instrument for determinants of innovations. Int J Qual Heal Care. 2014;26(5):501–10.

16. Drain PK, Dorward J, Violette LR, Quame-Amaglo J, Thomas KK, Samsunder N, et al. Point-of-care HIV viral load testing combined with task shifting to improve treatment outcomes (STREAM): findings from an open-label, non-inferiority, randomised controlled trial. Lancet HIV. 2020;7(4):e229–37.

17. Reif LK, Belizaire ME, Rouzier V, Seo G, Severe P, Bajo Joseph JM, et al. Point-of-care viral load testing among adolescents and young adults living with HIV in Haiti: a randomized control trial. AIDS Care - Psychol Socio-Medical Asp AIDS/HIV. 2022;34(4):409–20.

18. Nakyanzi A, Naddunga F, Bulterys MA, Mujugira A, Wyatt MA, Kamusiime B, et al. "It Soothes Your Heart": A Multimethod Study Exploring Acceptability of Point-of-Care Viral

Load Testing among Ugandan Pregnant and Postpartum Women Living with HIV. Diagnostics. 2024;14(1).
19. Boyce RM, Ndizeye R, Ngelese H, Baguma E, Shem B, Rubinstein RJ, et al. It takes more than a machine: A pilot feasibility study of point-of-care HIV-1 viral load testing at a lower-level health center in rural western Uganda. PLOS Glob Public Heal. 2023 Mar 27;3(3):e0001678.
20. Engel N, Davids M, Blankvoort N, Dheda K, Pant Pai N, Pai M. Making HIV testing work at the point of care in South Africa: A qualitative study of diagnostic practices. BMC Health Serv Res. 2017;17(1).
21. Drain PK, Dorward J, Violette LR, Quame-Amaglo J, Thomas KK, Samsunder N, et al. Point-of-care HIV viral load testing combined with task shifting to improve treatment outcomes (STREAM): findings from an open-label, non-inferiority, randomised controlled trial. Lancet HIV [Internet]. 2020;7(4):e229–37. Available from: http://dx.doi.org/10.1016/S2352-3018(19)30402-3
22. Ndlovu Z, Ellman T. Lay testing cadres and point-of-care diagnostic tests for HIV and other diseases: An essential combination in health service delivery. PLoS Med. 2021 Nov 1;18(11).
23. Mnzava D, Okuma J, Ndege R, Kimera N, Ntamatungiro A, Nyuri A, et al. Decentralization of viral load testing to improve HIV care and treatment cascade in rural Tanzania: observational study from the Kilombero and Ulanga Antiretroviral Cohort. BMC Infect Dis. 2023;23(1):1–11.
24. Boeke CE, Joseph J, Atem C, Banda C, Coulibaly KD, Doi N, et al. Evaluation of near point-of-care viral load implementation in public health facilities across seven countries in sub-Saharan Africa. J Int AIDS Soc. 2021;24(1):1–9.
25. Wang Y, Wagner AD, Liu S, Kingwara L, Oyaro P, Brown E, et al. Using queueing models as a decision support tool in allocating point-of-care HIV viral load testing machines in Kisumu County, Kenya. Health Policy Plan. 2024;39(1):44–55.
26. Rosen JG, Ddaaki WG, Nakyanjo N, Chang LW, Vo A Van, Zhao T, et al. The potential promise and pitfalls of point-of-care viral load monitoring to expedite HIV treatment decision-making in rural Uganda: a qualitative study. BMC Health Serv Res. 2024;24(1).

Supporting information

S1 Table. Items and constructs of the adapted MIDI for near point of care HIV VL monitoring

S2 Table.Demographic characteristics of seventy-five healthcare workers, including their division of interviews before, one month and six months of POC implementation, with some HCWs interviewed at more than one time point
