# Supplementary material for: Implementation bottlenecks of near point of care HIV viral load monitoring for children and young people in Tanzania: A Qualitative Study
Source: PLoS One. 2026 Jun 12;21(6):e0351304. doi: 10.1371/journal.pone.0351304 (PMC13262835; doi:10.1371/journal.pone.0351304)
Supplement: Attachment — Submitted filename: Comments.docx [file pone.0351304.s007.docx]

Manuscript Title: “Clients don’t want to wait two hours for their results!’ Healthcare workers’ implementation bottlenecks of near point-of-care viral load monitoring for children, adolescents, and young people with HIV in Tanzania: A qualitative study”

Summary

This manuscript presents a qualitative investigation of implementation bottlenecks in near point-of-care (nPOC) HIV viral load (VL) monitoring for children, adolescents, and young people living with HIV in Tanzania. The topic is highly relevant to global HIV program implementation, particularly in low- and middle-income countries (LMIC) settings where turnaround time, workforce capacity, and infrastructure remain persistent barriers to optimal HIV care.

The study is embedded within the broader EAPOC cluster-randomised trial and applies the Measurement Instrument for Determinants of Innovations (MIDI) framework to explore healthcare workers’ (HCWs’) experiences. The manuscript is generally well written, ethically sound, and methodologically appropriate for its stated aims. The findings are plausible, policy-relevant, and aligned with existing literature.

However, the paper would benefit from **greater analytical depth, more explicit linkage between qualitative findings and implementation outcomes,** and **stronger methodological transparency**, particularly regarding rigor, reflexivity, and triangulation. Several revisions are recommended before the manuscript is suitable for publication.

Major Comments

1. The objectives stated in the manuscript do not clearly articulate how both objectives are addressed analytically in the results section. The findings are presented descriptively, but the linkage to the two stated objectives, 1) factors contributing to implementation, and 2) challenges faced by HCWs, is not consistently explicit.

Recommendations: consider adding a brief paragraph at the beginning of the results section to clarify how the themes correspond to the objectives and MIDI domains.

2. The analysis provided is descriptive, while the MIDI framework is appropriate. Still, the manuscript does not sufficiently explain the listing facilitators and barriers to interpret, why specific determinants were more influential than others, or how they interact.

Recommendation: Explicitly discuss interconnections between MIDI domains (e.g., how organizational resource shortages undermine provider self-efficacy

3. Although the qualitative study is nested within the EAPOC trial, the manuscript does not sufficiently leverage this design strength. The relationship between qualitative findings and trial outcomes (e.g., viral suppression, turnaround time) remains implicit.

Recommendation: Integrate available quantitative indicators from the EAPOC trial (even descriptively) to contextualize qualitative findings. And, this mixed-methods triangulation would significantly strengthen the manuscript’s contribution.

4. In the sample strategy and saturation, although saturation is mentioned, the justification remains limited. The rationale for interviewing additional participants beyond the initial plan (from 60 to 75) is not fully explained.

Recommendation: Define how saturation was assessed, specify whether saturation was reached across all MIDI domains or only dominant ones. Clarify whether saturation differed by time point (baseline vs. post-implementation).

Minor Comments:

1. Title: The title is compelling but long; consider shortening while retaining the key message.
2. Abstract can be modified a bit; it should explicitly mention the MIDI framework
3. Consider adding a subheading aligned with MIDI domains to improve readability and ensure that all tables are explicitly referenced in the text.
4. Clarify more on how the MIDI tool was adapted specifically for the Tanzanian HIV context, indicating whether any domains were excluded or merged.

Decision: Major Revision

This manuscript addresses a vital implementation challenge in HIV care and is grounded in a sound qualitative framework. With revisions to strengthen analytical depth and methodological transparency, the study has strong potential to make a valuable contribution to the literature on point-of-care diagnostics and HIV service delivery.
