# Supplementary material for: Implementation bottlenecks of near point of care HIV viral load monitoring for children and young people in Tanzania: A Qualitative Study
Source: PLoS One. 2026 Jun 12;21(6):e0351304. doi: 10.1371/journal.pone.0351304 (PMC13262835; doi:10.1371/journal.pone.0351304)
Supplement: Peer review history

# Peer review: Decision Letter 0

**DOI:** 10.1371/journal.pone.0351304.r001

**Authors:** Hamufare Mugauri

5 Jan 2026

PONE-D-25-65447

‘Clients don’t want to wait two hours for their results!’ Healthcare workers’ Implementation Bottlenecks of Near Point of Care Viral Load Monitoring for Children, Adolescents, and Young People with HIV in Tanzania: A Qualitative Study

PLOS One

Dear Dr.  Msoka,

Thank you for submitting your manuscript to PLOS ONE. After careful consideration, we feel that it has merit but does not fully meet PLOS ONE’s publication criteria as it currently stands. Therefore, we invite you to submit a revised version of the manuscript that addresses the points raised during the review process.

Please submit your revised manuscript by Feb 19 2026 11:59PM. If you will need more time than this to complete your revisions, please reply to this message or contact the journal office at plosone@plos.org. When you're ready to submit your revision, log on to https://www.editorialmanager.com/pone/ and select the 'Submissions Needing Revision' folder to locate your manuscript file.

Please include the following items when submitting your revised manuscript:

- A letter that responds to each point raised by the academic editor and reviewer(s). You should upload this letter as a separate file labeled 'Response to Reviewers'.
- A marked-up copy of your manuscript that highlights changes made to the original version. You should upload this as a separate file labeled 'Revised Manuscript with Track Changes'.
- An unmarked version of your revised paper without tracked changes. You should upload this as a separate file labeled 'Manuscript'.

If you would like to make changes to your financial disclosure, please include your updated statement in your cover letter. Guidelines for resubmitting your figure files are available below the reviewer comments at the end of this letter.

If applicable, we recommend that you deposit your laboratory protocols in protocols.io to enhance the reproducibility of your results. Protocols.io assigns your protocol its own identifier (DOI) so that it can be cited independently in the future. For instructions see: https://journals.plos.org/plosone/s/submission-guidelines#loc-laboratory-protocols. Additionally, PLOS ONE offers an option for publishing peer-reviewed Lab Protocol articles, which describe protocols hosted on protocols.io. Read more information on sharing protocols at https://plos.org/protocols?utm_medium=editorial-email&utm_source=authorletters&utm_campaign=protocols.

We look forward to receiving your revised manuscript.

Kind regards,

Hamufare Dumisani Mugauri, Ph.D. Medicine and Health Sciences

Academic Editor

PLOS One

Journal Requirements:

When submitting your revision, we need you to address these additional requirements.

1.Please ensure that your manuscript meets PLOS ONE's style requirements, including those for file naming. The PLOS ONE style templates can be found at

https://journals.plos.org/plosone/s/file?id=wjVg/PLOSOne_formatting_sample_main_body.pdf and

https://journals.plos.org/plosone/s/file?id=ba62/PLOSOne_formatting_sample_title_authors_affiliations.pdf

2. Please note that funding information should not appear in any section or other areas of your manuscript. We will only publish funding information present in the Funding Statement section of the online submission form. Please remove any funding-related text from the manuscript.

3. We note that your Data Availability Statement is currently as follows: “All relevant data are within the manuscript and its Supporting Information files.”

Please confirm at this time whether or not your submission contains all raw data required to replicate the results of your study. Authors must share the “minimal data set” for their submission. PLOS defines the minimal data set to consist of the data required to replicate all study findings reported in the article, as well as related metadata and methods (https://journals.plos.org/plosone/s/data-availability#loc-minimal-data-set-definition).

For example, authors should submit the following data:

- The values behind the means, standard deviations and other measures reported;

- The values used to build graphs;

- The points extracted from images for analysis.

Authors do not need to submit their entire data set if only a portion of the data was used in the reported study.

If your submission does not contain these data, please either upload them as Supporting Information files or deposit them to a stable, public repository and provide us with the relevant URLs, DOIs, or accession numbers. For a list of recommended repositories, please see https://journals.plos.org/plosone/s/recommended-repositories.

If there are ethical or legal restrictions on sharing a de-identified data set, please explain them in detail (e.g., data contain potentially sensitive information, data are owned by a third-party organization, etc.) and who has imposed them (e.g., an ethics committee). Please also provide contact information for a data access committee, ethics committee, or other institutional body to which data requests may be sent. If data are owned by a third party, please indicate how others may request data access.

4. When completing the data availability statement of the submission form, you indicated that you will make your data available on acceptance. We strongly recommend all authors decide on a data sharing plan before acceptance, as the process can be lengthy and hold up publication timelines. Please note that, though access restrictions are acceptable now, your entire data will need to be made freely accessible if your manuscript is accepted for publication. This policy applies to all data except where public deposition would breach compliance with the protocol approved by your research ethics board. If you are unable to adhere to our open data policy, please kindly revise your statement to explain your reasoning and we will seek the editor's input on an exemption. Please be assured that, once you have provided your new statement, the assessment of your exemption will not hold up the peer review process.

5. Your ethics statement should only appear in the Methods section of your manuscript. If your ethics statement is written in any section besides the Methods, please move it to the Methods section and delete it from any other section. Please ensure that your ethics statement is included in your manuscript, as the ethics statement entered into the online submission form will not be published alongside your manuscript.

6. We note you have included a table to which you do not refer in the text of your manuscript. Please ensure that you refer to Table 2 in your text; if accepted, production will need this reference to link the reader to the Table.

7. If the reviewer comments include a recommendation to cite specific previously published works, please review and evaluate these publications to determine whether they are relevant and should be cited. There is no requirement to cite these works unless the editor has indicated otherwise.

Additional Editor Comments:

Title

- Current title includes a quotation and is lengthy. PLOS ONE prefers concise, descriptive titles without quotes.
- Suggested revision:
*Implementation Bottlenecks of Near Point-of-Care HIV Viral Load Monitoring for Children and Adolescents in Tanzania: A Qualitative Study*

Abstract

- Needs explicit mention of sample size and key findings in numerical terms.
- Keywords should be standardized and separated by semicolons.

Introduction

- Well-written but could be condensed to avoid excessive background detail.
- Ensure UNAIDS and WHO statistics are cited with proper hyperlinks or DOI.

Methods

- Must include study registration details if applicable (e.g., clinical trial registration).
- Clarify whether saturation was predefined and how it was assessed.
- Provide more detail on NVivo coding process for reproducibility.

Results

- Tables should follow PLOS ONE formatting (no merged cells, clear captions).
- Quotes from participants should be anonymized consistently (e.g., “Doctor, female, 42 years” instead of “Doctor, 42 yrs, female, T2”).

Discussion

- Needs clearer linkage to global literature and implications for scale-up.
- Avoid redundancy with Results section.

Data Availability

- PLOS ONE requires public data sharing in a repository (e.g., Dryad, Figshare) unless restricted by ethics. Current statement (“available upon signing a data transfer agreement”) does not meet PLOS ONE’s open data policy.

Formatting

- Ensure compliance with PLOS ONE style:Use SI units consistently.
- Remove underlining and hyperlinks in author details.
- References should follow PLOS ONE format (numbered, with DOI where possible).
  Language and Clarity
- Minor grammatical issues and long sentences need editing for clarity.
- Avoid colloquial expressions (e.g., “Clients don’t want to wait two hours…” in title).

[Note: HTML markup is below. Please do not edit.]

Reviewer's Responses to Questions

**Comments to the Author**

1. Is the manuscript technically sound, and do the data support the conclusions?

The manuscript must describe a technically sound piece of scientific research with data that supports the conclusions. Experiments must have been conducted rigorously, with appropriate controls, replication, and sample sizes. The conclusions must be drawn appropriately based on the data presented.

Reviewer #1: Partly

Reviewer #2: Yes

Reviewer #3: Yes

**********

2. Has the statistical analysis been performed appropriately and rigorously?

Reviewer #1: N/A

Reviewer #2: Yes

Reviewer #3: No

**********

3. Have the authors made all data underlying the findings in their manuscript fully available?

The PLOS Data policy requires authors to make all data underlying the findings described in their manuscript fully available without restriction, with rare exception (please refer to the Data Availability Statement in the manuscript PDF file). The data should be provided as part of the manuscript or its supporting information, or deposited to a public repository. For example, in addition to summary statistics, the data points behind means, medians and variance measures should be available. If there are restrictions on publicly sharing data—e.g. participant privacy or use of data from a third party—those must be specified.

Reviewer #1: No

Reviewer #2: Yes

Reviewer #3: Yes

**********

4. Is the manuscript presented in an intelligible fashion and written in standard English?

PLOS ONE does not copyedit accepted manuscripts, so the language in submitted articles must be clear, correct, and unambiguous. Any typographical or grammatical errors should be corrected at revision, so please note any specific errors here.

Reviewer #1: No

Reviewer #2: Yes

Reviewer #3: Yes

**********

5. Review Comments to the Author

Please use the space provided to explain your answers to the questions above. You may also include additional comments for the author, including concerns about dual publication, research ethics, or publication ethics. (Please upload your review as an attachment if it exceeds 20,000 characters)

Reviewer #1: The author should use the COREQ checklist to address the missing items and improve the work flow. The author should also revise the methods sections,specifically the data collection procedure.

Revise the result section, the structure is somehow confusing, themes and subthemes are not clearly presented

Consider proof reading the work (language editing), some sentences could be revised and merged to improve clarity

Reviewer #2: 1. Can you clarify: Line 259 of the PDF proof: the VL figure is >100copies/ml? the correct figure should be >1000copies/ml

2. Results section: can you organize the responses by subheads? e.g Factors facilitating implementation of PoC:

i. Personal benefit

ii. Professional obligation

iii. Completeness

Also follow similar pattern for Challenges in implementing nPOC HIV VL monitoring

Reviewer #3: Major Comments

1. The objectives stated in the manuscript do not clearly articulate how both objectives are addressed analytically in the results section. The findings are presented descriptively, but the linkage to the two stated objectives, 1) factors contributing to implementation, and 2) challenges faced by HCWs, is not consistently explicit.

Recommendations: consider adding a brief paragraph at the beginning of the results section to clarify how the themes correspond to the objectives and MIDI domains.

2. The analysis provided is descriptive, while the MIDI framework is appropriate. Still, the manuscript does not sufficiently explain the listing facilitators and barriers to interpret, why specific determinants were more influential than others, or how they interact.

Recommendation: Explicitly discuss interconnections between MIDI domains (e.g., how organizational resource shortages undermine provider self-efficacy

3. Although the qualitative study is nested within the EAPOC trial, the manuscript does not sufficiently leverage this design strength. The relationship between qualitative findings and trial outcomes (e.g., viral suppression, turnaround time) remains implicit.

Recommendation: Integrate available quantitative indicators from the EAPOC trial (even descriptively) to contextualize qualitative findings. And, this mixed-methods triangulation would significantly strengthen the manuscript’s contribution.

4. In the sample strategy and saturation, although saturation is mentioned, the justification remains limited. The rationale for interviewing additional participants beyond the initial plan (from 60 to 75) is not fully explained.

Recommendation: Define how saturation was assessed, specify whether saturation was reached across all MIDI domains or only dominant ones. Clarify whether saturation differed by time point (baseline vs. post-implementation).

Minor Comments:

1. Title: The title is compelling but long; consider shortening while retaining the key message.

2. Abstract can be modified a bit; it should explicitly mention the MIDI framework

3. Consider adding a subheading aligned with MIDI domains to improve readability and ensure that all tables are explicitly referenced in the text.

4. Clarify more on how the MIDI tool was adapted specifically for the Tanzanian HIV context, indicating whether any domains were excluded or merged.

Decision: Major Revision

This manuscript addresses a vital implementation challenge in HIV care and is grounded in a sound qualitative framework. With revisions to strengthen analytical depth and methodological transparency, the study has strong potential to make a valuable contribution to the literature on point-of-care diagnostics and HIV service delivery.

**********

6. PLOS authors have the option to publish the peer review history of their article (what does this mean?). If published, this will include your full peer review and any attached files.

If you choose “no”, your identity will remain anonymous but your review may still be made public.

**Do you want your identity to be public for this peer review?** For information about this choice, including consent withdrawal, please see our Privacy Policy.

Reviewer #1: No

Reviewer #2: No

Reviewer #3: No

**********

[NOTE: If reviewer comments were submitted as an attachment file, they will be attached to this email and accessible via the submission site. Please log into your account, locate the manuscript record, and check for the action link "View Attachments". If this link does not appear, there are no attachment files.]

To ensure your figures meet our technical requirements, please review our figure guidelines: https://journals.plos.org/plosone/s/figures.

You may also use PLOS’s free figure tool, NAAS, to help you prepare publication quality figures: https://journals.plos.org/plosone/s/figures#loc-tools-for-figure-preparation

NAAS will assess whether your figures meet our technical requirements by comparing each figure against our figure specifications.

Attachment Submitted filename: PONE-D-25-65447_reviewer.pdf

Attachment Submitted filename: Comments.docx

# Author comment: Author response to Decision Letter 1

**DOI:** 10.1371/journal.pone.0351304.r002

12 Mar 2026

Re: Response manuscript PONE-D-25-65447

We sincerely thank you for your valuable feedback and insightful comments, which have helped us revise the manuscript. We appreciate the time and effort you dedicated to reviewing our work. Below, we outline your comments along with our corresponding responses:

Additional Editor Comments:

Title

• Current title includes a quotation and is lengthy. PLOS ONE prefers concise, descriptive titles without quotes.

Response: We thank the editor for the comment. We have shortened the title to:

Implementation Bottlenecks of Near Point-of-Care HIV Viral Load Monitoring for Children and Young People in Tanzania: A Qualitative Study

Abstract

• Needs explicit mention of sample size and key findings in numerical terms.

Response: We thank the Editor for the comment. We have mentioned the sample size in the methods section and key findings in numerical terms. page 2 Lines 39-40 and 47-49 as:

“We purposively selected 75 HCWs involved in implementing nPOC at the intervention sites for in-depth interviews.”

A total of 75 HCWs participated across three time points (months 0, 6, and 12), which enabled demonstration of gains in knowledge, confidence, and adherence to procedures after training.”

• Keywords should be standardized and separated by semicolons.

Response: We thank the Editor for the comment. We have made changes to the keywords in a standardised format with semicolon lines. Page 3 lines 60-61 as:

“Keywords: HIV; point of care viral load testing; implementation bottlenecks; children; young people; healthcare workers”

Introduction

• Well-written but could be condensed to avoid excessive background detail.

Response: We thank the Editor for the comment. We have revised and condensed the introduction section accordingly. Page 4-5, lines 77-115

• Ensure UNAIDS and WHO statistics are cited with proper hyperlinks or DOI.

Response: We thank the Editor for the comment, the UNAIDS and WHO statistics are cited with proper hyperlinks or DOI

Methods

• Must include study registration details if applicable (e.g., clinical trial registration).

Response: We thank the Editor for the comment. We have included the study registration details on page4, line 104. Study Registration NCT05048472.

• Clarify whether saturation was predefined and how it was assessed.

Response: We thank the Editor for the comment. The clarity of whether saturation was predefined and how it was assessed is explained well on page 7, lines 147-152 and page 11, lines 185-191 as:

“To guide our sampling, we planned to conduct 60 interviews, based on prior qualitative research indicating that this sample size is generally sufficient to reach data saturation; this would have meant approximately four interviews per site per time point.[14,15]To ensure adequate representation and achieve a minimum of five HCWs per site at each time point, the total number of interviews was increased to 75, providing full coverage of all four domains of the Measuring Instrument for Determinants of Innovations (MIDI).

Saturation was assessed by continuously reviewing the data throughout the interview process to determine whether new themes were emerging. While saturation was reached across most of the MIDI domains, the themes varied slightly by time point: baseline interviews focused more on provider knowledge, self-efficacy, and compatibility with existing practices, while post-implementation interviews introduced new perspectives on organisational coordination, workflow, and client responses to nPOC HIV VL testing. At month six of the interviews, no new themes emerged, indicating that full saturation had been achieved across sites and domains.”

• Provide more detail on the NVivo coding process for reproducibility.

Response: We thank the Editor for the comment. More detailed explanation of NVivo coding process has been added to the data analysis section, page 12, lines 213-222 as:

“We conducted a deductive thematic framework analysis using the MIDI tool, with an inductive approach added. All transcripts were imported into NVivo 12 for coding and organisation. Memos were created by two researchers (PM and AM) for the first six transcripts, which were coded using a preliminary codebook based on the MIDI framework. The results, including potential new subthemes, were discussed with the last author (MSB), who provided guidance and helped refine the coding framework. The combined deductive–inductive coding framework was then applied by PM to the remaining 69 transcripts. We used NVivo 12 to organise the codes into nodes, highlighting them with coding stripes for consistency, and to track analytic decisions via memos, making the process clear and easy to follow. We created a summary table linking codes to quotes across the four MIDI domains to clearly show how the data aligned with the framework.”

Results

• Tables should follow PLOS ONE formatting (no merged cells, clear captions).

Response: We appreciate the reviewer's comment. We have updated the format of tables 1 and 2 to meet PLOS One requirements.

• Quotes from participants should be anonymized consistently (e.g., “Doctor, female, 42 years” instead of “Doctor, 42 yrs, female, T2”).

Response: We thank the Editor for the comment. We have made changes to the quotes for consistency on pages 16-22, lines 300-481

Discussion

• Needs clearer linkage to global literature and implications for scale-up.

Response: We appreciate the reviewer's comment and have made the corresponding changes to the discussion section on page 25. Lines 571, and Lines 601-607 as:

“Globally, studies have shown that countries with limited resources face similar challenges in implementing nPOC HIV VL monitoring, including staff shortages, weak policies, and supply problems.[10,26,27] This information confirms that HIV testing and the ability to successfully conduct HIV VL tests are global issues that affect large parts of the world, not just Tanzania.

To scale up nPOC HIV VL monitoring, efforts should be made to improve access to HIV VL testing, including training the individuals who conduct these tests, improving supply chains, and integrating nPOC services into national laboratory systems. Studies from Malawi and South Africa demonstrate that implementing strong policies, conducting regular staff training, and providing supervisory support enhance both programme sustainability and results.[26,28] Therefore, the scaling up of interventions in Tanzania cannot be viewed solely as an effort to expand access to a technology, but must also be seen as part of an overall investment in strengthening the entire health system.”

• Avoid redundancy with Results section.

Response: We appreciate the reviewer's feedback. We have revised the discussion section to minimise overlap with the results on pages 24-26, lines 520-604.

Data Availability

• PLOS ONE requires public data sharing in a repository (e.g., Dryad, Figshare) unless restricted by ethics. Current statement (“available upon signing a data transfer agreement”) does not meet PLOS ONE’s open data policy.

Response: We thank the reviewer for the comment. We have revised the Data Availability statement to clarify the data access conditions in accordance with Tanzania's national regulations. The updated statement now reads as follows:

“Data cannot be made publicly available because national regulations in Tanzania only allow data sharing with an approved Data Transfer Agreement (DTA). Access to the data requires authorization from the relevant authorities in Tanzania and signing of the DTA by the data receiver. Tanzania is currently developing a national data repository where metadata from this study will be shared once available. The full dataset is available upon reasonable request at Kilimanjaro Clinical Research Institute (KCRI), Moshi, Tanzania.”

Formatting

• Ensure compliance with PLOS ONE style:

o Use SI units consistently.

o Remove underlining and hyperlinks in author details.

o References should follow PLOS ONE format (numbered, with DOI where possible).

Response: We thank the reviewer for the comment. We have revised the manuscript accordingly.SI units are now used consistently throughout the text, underlining and hyperlinks have been removed from the author details, and all references have been reformatted to follow the PLOS One style( numbered and including DOIs where available)

Language and Clarity

• Minor grammatical issues and long sentences need editing for clarity.

• Avoid colloquial expressions (e.g., “Clients don’t want to wait two hours…” in title).

Response: We thank the reviewer for the comment. We have revised long sentences and edited for clarity. We have also updated the title by removing the quote.

Reviewer #1: The author should use the COREQ checklist to address the missing items and improve the workflow.

Response: We thank the reviewer for this helpful suggestion. The manuscript has been revised in accordance with the COREQ checklist to improve reporting transparency and workflow. Specifically, we have (1) described the continuous assessment of data saturation across the different time points; (2) improved transparency of the coding process by adding analytic memos, coding stripes and summary tables; (3) clarified participant flow, stating that no participants refused participation or dropped out and that no repeat interviews were conducted; (4) specified that none of the interviewers had a prior relationship with the participants; (5) clarified the interview setting, privacy conditions and consent procedures; and (6) expanded the description of themes and subthemes in the result section.

The author should also revise the methods section, specifically the data collection procedure.

Response: We thank the reviewer for the comment. The data collection procedure has been revised to address the missing items on page 11, lines 178-191, as follows:

“Before starting the interviews, we provided all HCWs with a thorough explanation of the study, including its objectives and procedures, and obtained written informed consent. The interviews were conducted by trained researchers with backgrounds in qualitative research. Both researchers had prior experience working with HCWs in HIV care settings and none had a prior relationship with the participants before recruitment. We interviewed HCWs at three time points (months 0, 6, and 12). No repeat interviews were conducted with participants; each participant was interviewed once at each time point.

Saturation was assessed by continuously reviewing the data throughout the interview process to determine whether new themes were emerging. While saturation was reached across most of the MIDI domains, the themes varied slightly by time point: baseline interviews focused more on provider knowledge, self-efficacy, and compatibility with existing practices, while post-implementation interviews introduced new perspectives on organisational coordination, workflow, and client responses to nPOC HIV VL testing. At month six of the interviews, no new themes emerged, indicating that full saturation had been achieved across sites and domains.”

Revise the result section, the structure is somehow confusing, themes and subthemes are not clearly presented

Response: We thank the reviewer for the comment. The results are presented in alignment with the study objectives: (1) to investigate factors that contributed to the successful implementation of nPOC HIV VL monitoring and (2) to explore challenges faced by HCWs during implementation. The implementation process of nPOC HIV VL monitoring was guided by the MIDI framework, with findings organised into themes mapped across its four domains. Within each theme, subthemes were identified based on the 29 MIDI subdomains.

Consider proof reading the work (language editing), some sentences could be revised and merged to improve clarity

Response: We thank the reviewer for the comment. We have proofread and revised the sentences for clarity accordingly.

Reviewer #2: 1. Can you clarify: Line 259 of the PDF proof: the VL figure is >100copies/ml? the correct figure should be >1000copies/ml

Response: We thank the reviewer for the comment. We have made changes in page 15, line 292 to correct the figure: (≥ 1000 copies/mL).

2. Results section: can you organize the responses by subheads? e.g. Factors facilitating implementation of PoC:

i. Personal benefit

ii. Professional obligation

iii. Completeness

Also follow similar pattern for Challenges in implementing nPOC HIV VL monitoring

Response: We thank the reviewer for the comment. We have made changes to the result section accordingly, pages 14-23, lines 255-515.

Reviewer #3: Major Comments

1. The objectives stated in the manuscript do not clearly articulate how both objectives are addressed analytically in the results section. The findings are presented descriptively, but the linkage to the two stated objectives, 1) factors contributing to implementation, and 2) challenges faced by HCWs, is not consistently explicit.

Recommendations: consider adding a brief paragraph at the beginning of the results section to clarify how the themes correspond to the objectives and MIDI domains.

Response: We thank the reviewer for the comment. We have added the paragraph at the beginning of the results section to clarify how the themes correspond to the objectives and MIDI domains, page 14, lines 256-264 as:

“The results are presented in alignment with the study objectives: (1) to investigate factors that contributed to the successful implementation of nPOC HIV VL monitoring and (2) to explore challenges faced by HCWs during implementation. The implementation process of nPOC HIV VL monitoring was guided by the MIDI framework, with findings organised into themes mapped across its four domains. Within each theme, subthemes were identified based on the 29 MIDI subdomains. To provide context for the findings, we first describe the socio-demographic characteristics of participants and the pre-implementation context. Finally, we describe the key facilitators and challenges in two sections, each organised by the MIDI domains.”

2. The analysis provided is descriptive, while the MIDI framework is appropriate. Still, the manuscript does not sufficiently explain the listing facilitators and barriers to interpret, why specific determinants were more influential than others, or how they interact.

Recommendation: Explicitly discuss interconnections between MIDI domains (e.g., how organizational resource shortages undermine provider self-efficacy

Response: We thank the reviewer for the comment. The interconnections between MIDI domains have been explained well in the discussion section, page 25-26, lines 575-582 as:

“The overall analysis has shown that there are important interactions between MIDI domains. While HCWs experienced the intervention as simple and compatible with the existing system, they were unable to use it effectively due to a lack of support from their organisations (particularly in terms of workforce and equipment). In addition, the lack of alignment between policy and guidelines (in both the social and political contexts) compounded organisational difficulties, leading to decreased confidence in providers’ abilities to implement the intervention effectively. These connections highlight that successful implementation depends on addressing not only individual and intervention-level determinants, but also barriers posed by organisations and the social and political context.”

3. Although the qualitative study is nested within the EAPOC trial, the manuscript does not sufficiently leverage this design strength. The relationship between qualitative findings and trial outcomes (e.g., viral suppression, turnaround time) remains implicit.

Recommendation: Integrate available quantitative indicators from the EAPOC trial (even descriptively) to contextualize qualitative findings. And, this mixed-methods triangulation would significantly strengthen the manuscript’s contribution.

Response: We thank the reviewer for this insightful comment. We would like to clarify that this study is entirely qualitative in design and purpose. The quantitative is beyond the scope of this manuscript. Furthermore, the quantitative r

# Editor report: Decision Letter 1

**DOI:** 10.1371/journal.pone.0351304.r003

**Authors:** Hamufare Mugauri

16 Mar 2026

PONE-D-25-65447R1

Implementation Bottlenecks of Near Point of Care HIV Viral Load Monitoring for Children and Young People in Tanzania: A Qualitative Study

PLOS One

Dear Dr. Msoka,

Thank you for submitting your manuscript to PLOS ONE. After careful consideration, we feel that it has merit but does not fully meet PLOS ONE’s publication criteria as it currently stands. Therefore, we invite you to submit a revised version of the manuscript that addresses the points raised during the review process.

Please submit your revised manuscript by Apr 30 2026 11:59PM. If you will need more time than this to complete your revisions, please reply to this message or contact the journal office at plosone@plos.org. When you're ready to submit your revision, log on to https://www.editorialmanager.com/pone/ and select the 'Submissions Needing Revision' folder to locate your manuscript file.

Please include the following items when submitting your revised manuscript:

- A letter that responds to each point raised by the academic editor and reviewer(s). You should upload this letter as a separate file labeled 'Response to Reviewers'.
- A marked-up copy of your manuscript that highlights changes made to the original version. You should upload this as a separate file labeled 'Revised Manuscript with Track Changes'.
- An unmarked version of your revised paper without tracked changes. You should upload this as a separate file labeled 'Manuscript'.

If you would like to make changes to your financial disclosure, please include your updated statement in your cover letter. Guidelines for resubmitting your figure files are available below the reviewer comments at the end of this letter.

If applicable, we recommend that you deposit your laboratory protocols in protocols.io to enhance the reproducibility of your results. Protocols.io assigns your protocol its own identifier (DOI) so that it can be cited independently in the future. For instructions see: https://journals.plos.org/plosone/s/submission-guidelines#loc-laboratory-protocols. Additionally, PLOS ONE offers an option for publishing peer-reviewed Lab Protocol articles, which describe protocols hosted on protocols.io. Read more information on sharing protocols at https://plos.org/protocols?utm_medium=editorial-email&utm_source=authorletters&utm_campaign=protocols.

We look forward to receiving your revised manuscript.

Kind regards,

Hamufare Dumisani Mugauri, Ph.D. Epidemiology and Public Health

Academic Editor

PLOS One

Journal Requirements:

If the reviewer comments include a recommendation to cite specific previously published works, please review and evaluate these publications to determine whether they are relevant and should be cited. There is no requirement to cite these works unless the editor has indicated otherwise.

Additional Editor Comments:

Your study addresses an important implementation question for nPOC HIV viral load (VL) services and is broadly suitable for PLOS ONE, which evaluates methodological soundness over perceived novelty. The qualitative design (interviews and observations using the MIDI framework) is appropriate and yields potentially actionable findings for service delivery. However, before the manuscript can proceed, several substantive issues must be resolved to meet PLOS ONE’s publication criteria and qualitative reporting expectations (COREQ/SRQR), and to comply with PLOS data‑sharing policy:

Essential revisions (must address in your resubmission)

1. Qualitative reporting—complete COREQ/SRQR compliance

o Please upload a completed COREQ (32 item) or SRQR (21 item) checklist and revise the text to cover missing items. In particular, add: (a) researcher characteristics/reflexivity; (b) recruitment flow and non participation; (c) whether and how interview guides evolved; (d) number of coders, double coding/consensus procedures, and a coding tree; (e) credibility strategies (e.g., triangulation with observations, peer debriefing, member checking if used); and (f) clearer identifiers for quotations (cadre, gender, age) including dissenting or deviant cases. Include the interview guide(s) and observation checklist as supplementary files.

2. Internal consistency and numerical accuracy

o Repeat interviews: The Methods state “no repeat interviews,” yet the Results indicate that 17 of 25 participants at one month were followed up again at six months. Clarify the design (cross sectional vs. longitudinal subset), and present a simple sampling/retention diagram showing numbers approached, consented, interviewed at each time point, and re interviewed.

o Table 2 counts: Recalculate and reconcile all totals and percentages (e.g., the “Doctors” category totals vs. stratum sums). Ensure consistent denominators across columns.

3. WHO viral load thresholds—correct policy language

o Revise statements implying WHO “set the threshold at 50 copies/mL.” WHO’s 2023 policy brief defines three categories: undetectable (not detected), suppressed (detected but ≤1000 copies/mL), and unsuppressed (>1000 copies/mL); many national programs use >1000 copies/mL to define virologic failure while <50 copies/mL is often used clinically to denote undetectable. Please correct the Background/Discussion accordingly.

4. Data Availability Statement (DAS) compliant with PLOS policy

o PLOS requires that the minimal dataset underlying the findings be publicly available at publication (values underpinning analyses/quotes with de identification, coding tree, and analytic memos as feasible). When ethical/legal constraints apply (e.g., Tanzanian DTA requirements), the DAS must name a Data Access Committee, provide a contact, and describe a transparent request pathway, rather than “available on reasonable request.” Repository deposition with controlled access is strongly preferred (e.g., OSF/Zenodo/institutional). Please revise the DAS and prepare the data and documentation accordingly.

5. Strengthen analysis transparency

o Expand the Analysis subsection: specify number of coders, whether double coding was performed, how disagreements were resolved, and how deductive (MIDI) and inductive themes were integrated (e.g., codebook evolution, matrices). Provide a coding tree as Supplement.

6. Clarify POC vs. nPOC implementation

o Throughout, clearly distinguish true POC (on site cartridge testing) from nPOC (nearby laboratory; includes transport by boda boda). Where transport to a nearby lab occurred, specify typical transport and queue times if available, and ensure the Discussion reflects these operational differences.

7. Trial registration and cross referencing

o Since this is a substudy of EAPOC VL, add the registry link (NCT05048472) in Methods for transparency and replication, and cite it in the first mention of the parent trial.

[Note: HTML markup is below. Please do not edit.]

To ensure your figures meet our technical requirements, please review our figure guidelines: https://journals.plos.org/plosone/s/figures.

You may also use PLOS’s free figure tool, NAAS, to help you prepare publication quality figures: https://journals.plos.org/plosone/s/figures#loc-tools-for-figure-preparation

NAAS will assess whether your figures meet our technical requirements by comparing each figure against our figure specifications.

# Author comment: Author response to Decision Letter 2

**DOI:** 10.1371/journal.pone.0351304.r004

21 May 2026

To: Editorial Manager

PLOS One Journal

Dear Editors,

Re: Response manuscript PONE-D-25-65447

We sincerely thank you for your valuable feedback and insightful comments, which have helped us revise the manuscript. We appreciate the time and effort you dedicated to reviewing our work. Below, we outline your comments along with our corresponding responses:

Additional Editor Comments:

Title

• Current title includes a quotation and is lengthy. PLOS ONE prefers concise, descriptive titles without quotes.

Response: We thank the editor for the comment. We have shortened the title to:

Implementation Bottlenecks of Near Point-of-Care HIV Viral Load Monitoring for Children and Young People in Tanzania: A Qualitative Study

Abstract

• Needs explicit mention of sample size and key findings in numerical terms.

Response: We thank the Editor for the comment. We have mentioned the sample size in the methods section and key findings in numerical terms. page 2, lines 39-40, page 3, lines 47-49 as:

“We purposively selected 25 HCWs involved in implementing POC at the intervention sites for in-depth interviews.

A total of 75 interviews were conducted among 43 HCWs across 3 time points: 33 at baseline (T0), 25 at month 1 (T1; 19 participants from T0 and 6 new participants) and 17 at month 6 follow up (T2;6 participants from T1, 7 returning participants from T0 and 4 new participants).

• Keywords should be standardized and separated by semicolons.

Response: We thank the Editor for the comment. We have made changes to the keywords in a standardised format with semicolon lines. Page 3 lines 59-60 as:

“Keywords: HIV; point-of- care viral load testing; implementation bottlenecks; children; young people; healthcare workers.

Introduction

• Well-written but could be condensed to avoid excessive background detail.

Response: We thank the Editor for the comment. We have revised and condensed the background section accordingly. Page 4-5, lines 68-110

Ensure UNAIDS and WHO statistics are cited with proper hyperlinks or DOI.

Response: We thank the Editor for the comment. The UNAIDS and WHO statistics are cited with proper hyperlinks or DOI

Methods

• Must include study registration details if applicable (e.g., clinical trial registration).

Response: We thank the Editor for the comment. We have included the study registration details on page 6, line 114-115, as “We conducted an exploratory qualitative study nested within the EAPOC-VL trial (ClinicalTrials.gov identifier: NCT05048472; https://clinicaltrials.gov/study/NCT05048472) from January 2023 to January 2024.

• Clarify whether saturation was predefined and how it was assessed.

Response: We thank the Editor for the comment. The clarity of whether saturation was predefined and how it was assessed is explained well on page 7, lines 145-151, page 12, lines 181-187 as:

We initially planned to conduct an average of 25 interviews (five per site) across T0, T1, and T2, for a total of 75 interviews based on saturation principle. All HCWs invited agreed to participate and no participant withdrew from the study. However, not all HCWs enrolled at baseline were available for follow-up interviews at T1 and T2 due to annual leave, staff transfers, and changing clinical responsibilities. To ensure sufficient data, new participants were recruited consistently with the study’s combined longitudinal and cross-sectional design.

Saturation was assessed by continuously reviewing the data throughout the interview process to determine whether new themes were emerging. While saturation was reached across most of the MIDI domains, the themes varied slightly by time point: baseline interviews focused more on provider knowledge, self-efficacy, and compatibility with existing practices, while post-implementation interviews introduced new perspectives on organisational coordination, workflow, and client responses to nPOC HIV VL testing. At month six of the interviews, no new themes emerged, indicating that full saturation had been achieved across sites and domains.

• Provide more detail on the NVivo coding process for reproducibility.

Response: We thank the Editor for the comment. More detailed explanation of NVivo coding process has been added to the data analysis section, pages 13-14, lines 217-228, as:

We conducted a deductive thematic framework analysis using the MIDI tool, with an inductive approach added. All transcripts were imported into NVivo 12 for coding and organisation. Two researchers (PM and AM) double coded the first six transcripts using a preliminary codebook based on the MIDI framework. During this phase, inductive codes were generated to capture data that did not fit within the redefined MIDI categories. Analytic memos were created during this stage and discrepancies were resolved through discussion and consensus leading to refinement of the coding framework. PM applied the refined combined deductive (MIDI-based) codes with inductively derived subthemes to the remaining 69 transcripts. For consistency, we repeatedly reread the transcripts during analysis and held ongoing discussions within the research team to confirm interpretation. We used NVivo 12 to organise the codes into nodes, highlighting them with coding stripes for consistency, and to track analytic decisions via memos, making the process clear and easy to follow. We created a summary table linking codes to quotes across the four MIDI domains to clearly show how the data aligned with the framework.

Results

• Tables should follow PLOS ONE formatting (no merged cells, clear captions).

Response: We appreciate the Editor’s comment. We have updated the format of tables 1 and 2 to meet PLOS One requirements.

• Quotes from participants should be anonymized consistently (e.g., “Doctor, female, 42 years” instead of “Doctor, 42 yrs, female, T2”).

Response: We thank the Editor for the comment. We have made changes to the quotes for consistency.

Discussion

• Needs clearer linkage to global literature and implications for scale-up.

Response: We appreciate the Editor's comment and have made the corresponding changes to the discussion section on page 32, Lines 621-625, page 33, lines 634-640.

Globally, studies have shown that countries with limited resources face similar challenges in implementing nPOC HIV VL monitoring, including staff shortages, weak policies, and supply problems. This information confirms that HIV testing and the ability to successfully conduct HIV VL testing and the ability to successfully conduct HIV VL tests are global issues that affect large parts of the world, not just Tanzania.

To scale up nPOC HIV VL monitoring, efforts should be made to improve access to HIV VL testing, including training individuals who conduct these tests, improving supply chains, and integrating nPOC services into national laboratory systems. Studies from Malawi and South Africa demonstrate that implementing strong policies, conducting regular staff training, and providing supervisory support enhance both programme sustainability and results. Therefore, the scaling up of interventions in Tanzania cannot be viewed solely as an effort to expand access to a technology but must also be seen as part of an overall investment in strengthening the entire health system.

• Avoid redundancy with Results section.

Response: We appreciate the Editors feedback. We have revised the discussion section to minimize overlap with the results. Page 30.

Data Availability

• PLOS ONE requires public data sharing in a repository (e.g., Dryad, Figshare) unless restricted by ethics. Current statement (“available upon signing a data transfer agreement”) does not meet PLOS ONE’s open data policy.

Response: We thank the Editor for the comment. We have revised the Data Availability statement as: Deidentified data sets used and/or analysed supporting each theme during the current study are available upon acceptance. Full audio recording, transcripts cannot be made publicly available because Tanzanian national regulations require a Data Transfer Agreement (DTA) approved by the Tanzania Commission for Science and Technology (COSTECH) and the National Institute for Medical Research (NIMR) prior to any data sharing. Currently, Tanzania is developing their own data repository where meta-data of the study will be shared soon. To gain access to the data, the receiver should contact Marion.sumari@gmail.com, who is the Principal Investigator of the EAPOC Project, or KCRI administrator (kcriadmin@kcri.ac.tz). The coding tree and quote code matrix are available as open supplementary materials with this article (Supplementary S3 Figure and S4 File). Page 35-36, lines 690-698

Formatting

• Ensure compliance with PLOS ONE style:

o Use SI units consistently.

o Remove underlining and hyperlinks in author details.

o References should follow PLOS ONE format (numbered, with DOI where possible).

Response: We thank the reviewer for the comment. We have revised the manuscript accordingly.SI units are now used consistently throughout the text, underlining and hyperlinks have been removed from the author details, and all references have been reformatted to follow the PLOS One style( numbered and including DOIs where available)

Language and Clarity

• Minor grammatical issues and long sentences need editing for clarity.

• Avoid colloquial expressions (e.g., “Clients don’t want to wait two hours…” in title).

Response: We thank the reviewer for the comment. We have revised long sentences and edited for clarity. Also, I have removed colloquial expressions in the title.

Your study addresses an important implementation question for nPOC HIV viral load (VL) services and is broadly suitable for PLOS ONE, which evaluates methodological soundness over perceived novelty. The qualitative design (interviews and observations using the MIDI framework) is appropriate and yields potentially actionable findings for service delivery. However, before the manuscript can proceed, several substantive issues must be resolved to meet PLOS ONE’s publication criteria and qualitative reporting expectations (COREQ/SRQR), and to comply with PLOS data sharing policy:

Essential revisions (must address in your resubmission)

1. Qualitative reporting—complete COREQ/SRQR compliance

o Please upload a completed COREQ (32 item) or SRQR (21 item) checklist and revise the text to cover missing items. In particular, add:

(a) researcher characteristics/reflexivity; (item 8)

Response: We thank the Editor for the comment. The interviews were conducted by trained researchers with backgrounds in qualitative research, comprising PM(MSW, PhD candidate), AM (MPH), RM (MPH), and RA (MD). All Tanzanian health researchers are familiar with HIV service contexts. All interviewers had prior experience working with HCWs in HIV care settings and none had a prior relationship with the participants before recruitment. Researcher reflexivity was considered throughout the study. PM, with a background in social science and PhD candidate, played a role in coordinating data collection. PM and other research assistants conducted clinic observations and in-depth interviews with HCWs at the selected sites. Then, PM led regular debriefing sessions with the research assistants for emerging themes and ensured the use of neutral probing language. HCWs were interviewed at three time points (months 0, 1 and 6). Page 11, lines 13, lines 171-180.

In addition, we have now added the COREQ checklist as a supplementary file.

(b) recruitment flow and non-participation (item 13)

Response: We thank the Editor for the comment. We initially planned to conduct an average of 25 interviews (five per site) across T0, T1, and T2, for a total of 75 interviews based on saturation principle. All HCWs invited agreed to participate and no participant withdrew from the study. However, not all HCWs enrolled at baseline were available for follow-up interviews at T1 and T2 due to annual leave, staff transfers, and changing clinical responsibilities. To ensure sufficient data, new participants were recruited consistently with the study’s combined longitudinal and cross-sectional design. Page 7, lines 145-151.

(c) whether and how interview guides evolved (Item 17);

Response: We conducted in-depth interviews (IDIs) lasting 30-45 minutes, using a semi-structured interview guide we initially developed based on the MIDI questionnaire by Fleuren et al., which identifies key factors influencing the implementation of an innovation. The guide was pilot tested by the research assistants to confirm its clarity and reliability before data collection began. The guide evolved across time points: baseline questions focused on prior knowledge, existing practices, and anticipated barriers; post-implementation prompts (months 1 and 6) were added by the research team (PM, AM, MSB) following team debriefings. These adaptations allowed to reflect emerging themes from early interviews and explore observed workflow changes, client responses, and sustainability concerns. To ensure the relevance of MIDI to the Tanzanian HIV service delivery context, the research team discussed and developed questions and prompts consistent with local terminology, health system structures, and HIV care practices in Tanzania. No domains were excluded or merged; rather, the language and examples used in the interview tools were contextualised to ensure HCWs could easily understand them. For example, constructs related to financial and material resources were expanded to capture recurrent supply chain constraints, electricity reliability, and infrastructure limitations frequently observed in Tanzanian clinics. However, descriptive and subjective norms proved difficult to operationalise in the Tanzanian context, hence the results were not informative and are not reported.

Data were also collected through structured observations in the clinics before and after nPOC HIV VL monitoring implementation using predefined checklists to systematically assess workflow, adherence to procedures, and consistency across sites. Informal observations were conducted concurrently to capture natural interactions, workflow adaptations, and contextual factors that might not be captured in the structured checklists. The final versions of all observation checklists and interview guides are provided in S2 Appendix. Pages 12-13, lines 189-210.

(d) number of coders, double coding/consensus procedures, and a coding tree; (Items 24-25)

Responses: We thank the editor for the comment. We have revised the coding procedure as follows: “Two researchers (PM and AM) double coded the first six transcripts using a preliminary codebook based on the MIDI framework. During this phase, inductive codes were generated to capture data that did not fit within the redefined MIDI categories. Analytic memos were created during this stage, and discrepancies were resolved through discussion and consensus, leading to refinement of the coding framework. PM applied the refined combined deductive (MIDI-based) codes with inductively derived subthemes to the remaining 69 transcripts. For consistency, we repeatedly reread the transcripts during analysis and held ongoing discussions within the research team to confirm interpretation. We used NVivo 12 to organise the codes into nodes, highlighting them with coding stripes for consistency, and to track analytic decisions via memos, making the process clear and easy to follow. We created a summary table linking codes to quotes across the four MIDI domains to clearly show how the data aligned with the framework. The full coding tree and a quote code matrix are provided as supplementary files, S3 Figure and S4 File, respectively.” Pages 13-14, line 218-228.

(e) credibility strategies (e.g., triangulation with observations, peer debriefing, member checking if used);

Responses: We thank the editor for the comment. “Credibility was enhanced through: (1) triangulation of IDIs with structured clinic observations; (2) peer debriefing with co-authors to make sure our interpretation makes sense; and (3) use of the MIDI framework as a guide to organise and check our coding of the data. Page 14, lines 229-231

and (f) clearer identifiers for quotations (cadre, gender, age) incl

# Editor report: Decision Letter 2

**DOI:** 10.1371/journal.pone.0351304.r005

**Authors:** Hamufare Mugauri

26 May 2026

Implementation Bottlenecks of Near Point of Care HIV Viral Load Monitoring for Children and Young People in Tanzania: A Qualitative Study

PONE-D-25-65447R2

Dear Dr. Msoka,

We’re pleased to inform you that your manuscript has been judged scientifically suitable for publication and will be formally accepted for publication once it meets all outstanding technical requirements.

Within one week, you’ll receive an e-mail detailing the required amendments. When these have been addressed, you’ll receive a formal acceptance letter and your manuscript will be scheduled for publication.

An invoice will be generated when your article is formally accepted. Please note, if your institution has a publishing partnership with PLOS and your article meets the relevant criteria, all or part of your publication costs will be covered. Please make sure your user information is up-to-date by logging into Editorial Manager at Editorial Manager® and clicking the ‘Update My Information' link at the top of the page. For questions related to billing, please contact billing support.

If your institution or institutions have a press office, please notify them about your upcoming paper to help maximize its impact. If they’ll be preparing press materials, please inform our press team as soon as possible -- no later than 48 hours after receiving the formal acceptance. Your manuscript will remain under strict press embargo until 2 pm Eastern Time on the date of publication. For more information, please contact onepress@plos.org.

Kind regards,

Hamufare Dumisani Mugauri, Ph.D. Epidemiology and Public Health

Academic Editor

PLOS One

Additional Editor Comments (optional):

Reviewers' comments:

# Editor report: Acceptance letter

**DOI:** 10.1371/journal.pone.0351304.r006

**Authors:** Hamufare Mugauri

PONE-D-25-65447R2

PLOS One

Dear Dr. Msoka,

I'm pleased to inform you that your manuscript has been deemed suitable for publication in PLOS One. Congratulations! Your manuscript is now being handed over to our production team.

At this stage, our production department will prepare your paper for publication. This includes ensuring the following:

* All references, tables, and figures are properly cited

* All relevant supporting information is included in the manuscript submission,

* There are no issues that prevent the paper from being properly typeset

You will receive further instructions from the production team, including instructions on how to review your proof when it is ready. Please keep in mind that we are working through a large volume of accepted articles, so please give us a few days to review your paper and let you know the next and final steps.

Lastly, if your institution or institutions have a press office, please let them know about your upcoming paper now to help maximize its impact. If they'll be preparing press materials, please inform our press team within the next 48 hours. Your manuscript will remain under strict press embargo until 2 pm Eastern Time on the date of publication. For more information, please contact onepress@plos.org.

You will receive an invoice from PLOS for your publication fee after your manuscript has reached the completed accept phase. If you receive an email requesting payment before acceptance or for any other service, this may be a phishing scheme. Learn how to identify phishing emails and protect your accounts at https://explore.plos.org/phishing.

If we can help with anything else, please email us at customercare@plos.org.

Thank you for submitting your work to PLOS ONE and supporting open access.

Kind regards,

PLOS ONE Editorial Office Staff

on behalf of

Dr Hamufare Dumisani Mugauri

Academic Editor

PLOS One
